# The effect of *LPA* Thr3888Pro on lipoprotein(a) and coronary artery disease is modified by the *LPA* KIV-2 variant 4925G>A

**DOI:** 10.1016/j.atherosclerosis.2022.04.023

**Published:** 2022-04-26

**Authors:** Rebecca Grüneis, Claudia Lamina, Silvia Di Maio, Sebastian Schönherr, Peter Zoescher, Lukas Forer, Gertraud Streiter, Annette Peters, Christian Gieger, Anna Köttgen, Florian Kronenberg, Stefan Coassin

**Affiliations:** a Institute of Genetic Epidemiology, Department of Genetics and Pharmacology, Medical University of Innsbruck, Austria; b German Center for Diabetes Research (DZD), München-Neuherberg, Germany; c Institute of Epidemiology, Helmholtz Zentrum München - German Research Center for Environmental Health, Neuherberg, Germany; d Research Unit of Molecular Epidemiology, Helmholtz Zentrum München, German Research Center for Environmental Health, Neuherberg, Germany; e Institute of Genetic Epidemiology, Faculty of Medicine and Medical Center, University of Freiburg, Freiburg, Germany and German Chronic Kidney Disease Study, Germany

**Keywords:** Lipoprotein(a), Kringle IV-2 repeat, Apolipoprotein(a), Coronary artery disease, Mutation, SNP interaction

## Abstract

**Background and aims:**

High lipoprotein(a) [Lp(a)] concentrations are associated with increased coronary artery disease (CAD) risk. Lp(a) is regulated mainly genetically by the *LPA* gene but involved genetic variants have not been fully elucidated. Improved understanding of the entanglements of genetic Lp(a) regulation may enhance genetic prediction of Lp(a) and CAD risk. We investigated an interaction between the well-known *LPA* missense SNP rs41272110 (known as Thr3888Pro) and the frequent *LPA* splicing mutation KIV-2 4925G>A.

**Methods:**

Effects on Lp(a) concentrations were investigated by multiple quantile regression in the German Chronic Kidney Disease (GCKD) study, KORA-F3 and KORA-F4 (n_total_ = 10,405) as well as in the UK Biobank (UKB) 200k exome dataset (n = 173,878). The impact of the interaction on CAD risk was assessed by survival analysis in UKB.

**Results:**

We observed a significant SNP-SNP interaction in all studies (*p* = 1.26e-05 to 3.03e-04). In quantile regression analysis, rs41272110 as a predictor shows no impact on Lp(a) (β = −0.06 [-0.79; 0.68], *p* = 0.879), but in a joint model including both SNPs as predictors, rs41272110 is associated with markedly higher Lp(a) (β = +9.40 mg/dL [6.45; 12.34], *p* = 4.07e-10). Similarly, rs41272110 shows no effect on CAD in UKB (HR = 1.01 [0.97; 1.04], *p* = 0.731), while rs41272110 carriers not carrying 4925G>A show an increased CAD risk (HR = 1.10 [1.04; 1.16], *p* = 6.9e-04). This group corresponds to 4% of the population. Adjustment for apolipoprotein(a) isoforms further modified the effect estimates markedly.

**Conclusions:**

This work emphasizes the complexity of the genetic regulation of Lp(a) and the importance to account for genetic subgroups in Lp(a) association studies and when interpreting genetic cardiovascular risk profiles.

## 1 Introduction

High lipoprotein(a) [Lp(a)] plasma concentrations are associated with increased risk of coronary artery disease (CAD), aortic valve stenosis, myocardial infarction, stroke and peripheral arterial disease [1–7]. The individual Lp(a) concentration is regulated mainly genetically by the *LPA* gene, which encodes for apolipoprotein(a) [apo(a)] [1]. Apo(a) consists of different kringle IV domains (KIV-1 to KIV-10) [1]. The KIV-2 domain is encoded in a copy number variation, which creates >40 apo(a) isoforms. These can be grouped into low molecular weight (LMW, 10–22 KIV) and high molecular weight isoforms (HMW, >22 KIV), with the former being associated with ≈4–5 times higher median Lp(a) than the latter [1]. The isoform size shows an inverse correlation with median Lp(a) concentrations and is the major genetic determinant of plasma Lp(a) [1]. Nevertheless, the individual Lp(a) concentrations can still vary by up to 200-fold within each isoform group [8,9]. However, causal variants that modify the effect of the isoforms have been elusive until recently [10–13]. The identification of such variants is exacerbated by the fact that many functional *LPA* SNPs are associated with defined isoform ranges, which can modify the true effects of SNPs [10,13–15].

*LPA* SNPs are also major contributors to genetic risk scores for Lp(a) and CAD [16]. However, non-linear relationships and SNP-SNP interactions have been described [11,14,17]. The study at hand focuses on a novel interaction between two well-known *LPA* SNPs: the missense variant rs41272110 (NP_005568.2: p.Thr1399Pro [18], also known as Thr3888Pro [19,20], Thr23Pro [20] or Thr12Pro [18]) and the splice site mutation KIV-2 4925G>A [10].

Rs41272110 [18] is a missense variant in the KIV-8 domain that is very frequent in Europeans (minor allele frequency (MAF) = 14.1%) and shows a pronounced MAF variance in other populations (MAF = 0%–18% in the 1000 Genomes project). It has been one of the first polymorphisms identified in the *LPA* gene and causes an amino acid substitution from threonine to proline. Proline is commonly regarded as an amino acid that induces conformational changes, which makes this variant suggestive for a functional impact. Thus the variant has repeatedly moved into the center of interest and has been implied in Lp (a) regulation [19–25]. However, no biochemical function has been conclusively identified. Prins et al. [18] proposed occurrence of a conformational change in apo(a), while others deemed it unlikely to be truly functional [22]. The effects on Lp(a) concentrations reported in early studies were inconsistent, with both increasing and decreasing effects (the key findings of these studies are summarized in Supplementary Table I) [19–21].

KIV-2 4925G>A is a frequent splice variant in the repetitive KIV-2 domain that has been identified as a major determinant of Lp(a) concentrations in the population [10,11,13]. It shows a MAF of ≈13% in Europeans (with a pronounced variability between populations [10], alike rs41272110) and reduces Lp(a) concentration by up to 31 mg/dL in LMW isoform carriers. It explains up to 19.3% of the Lp(a) variance [10] and is thus the SNP with the second strongest impact on isoform-adjusted Lp(a) variance [13].

We coincidentally noted that these two SNPs present very similar MAFs and occur in similar apo(a) isoform size ranges. Since we had reported previously that linkage disequilibrium (LD) patterns in *LPA* can modulate *LPA* SNP associations in unexpected manners [14,26], we hypothesized that rs41272110 and 4925G>A might be in partial LD and interact. The true effect of rs41272110 on Lp(a) and especially on CAD might thus be more complex than previously thought. We investigated this in 10,405 individuals from three study cohorts with detailed Lp(a) phenotypes available and further dissected the effect of both variants on CAD in the UK Biobank 200k exome sequencing dataset. This showed that the impact and effect direction of rs41272110 on CAD depends substantially on KIV-2 4925G>A.

## 2 Patients and methods

### 2.1 Study populations

Analyses were performed on 10,405 individuals from three study populations. Study population descriptions, baseline characteristics and MAFs of the investigated SNPs are given in Table 1 and in the Supplementary Methods. In brief, the German Chronic Kidney Disease (GCKD) study is a prospective cohort study. For this study, the cross-sectional part at the baseline examination was available for analysis, including 5,217 participants with moderate chronic kidney disease [27,28]. Lp(a) isoforms, Lp(a) concentrations and SNP data for rs41272110 and 4925G>A were available for 4,575 participants. The Cooperative Health Research in the Region of Augsburg (KORA) F3 and F4 [29] are population-based studies including 3,184, respectively 3,080 participants. Lp(a) isoforms, Lp(a) concentrations and SNP data for both variants were available for 2,939 participants in KORA F3 and for 2,891 participants in KORA F4.

UK Biobank (UKB) [30] is a large-scale prospective study including more than 500,000 individuals aged 40–69 at recruitment (2006–2010). Analyses were restricted to 186,088 individuals of European ancestry (British, Irish or any other white European background) with available whole exome sequencing (for KIV-2 4925G>A) and whole genome genotyping data (for rs41272110). To investigate the impact of KIV-2 4925G>A and rs41272110 on Lp(a) concentrations, data were further restricted to participants with available Lp(a) measurements (n = 173,878).

For all studies written informed consent was obtained from each individual and the study protocol has been approved by the responsible ethics committees. The study protocols conform to the Declaration of Helsinki.

### 2.2 Lp(a) phenotyping

In GCKD, KORA F3 and KORA F4 Lp(a) concentrations were determined by ELISA in mg/dL. A polyclonal affinity-purified rabbit anti-human apo(a) antibody was used for coating and a horseradish peroxidase-conjugated monoclonal anti-apo(a) antibody 1A2 for detection [31]. Apo(a) isoforms were assessed by Western blotting (Supplementary Methods) [32,33]. Both variables were available from previous projects and a detailed protocol for ELISA and Western blotting has been published in [32]. All ELISA and Western blot data were measured with the same assay and scored by the same experienced researcher. Isoform designations report the total number of KIV domains.

In the UK Biobank (UKB) Lp(a) concentration was measured using an immunoturbidimetric assay (Randox Laboratories; Crumlin, County Antrim, United Kingdom using a Beckman Coulter AU5800 Platform), with clinically reportable range 3.8–189 nmol/L. 11.04% and 7.05% of individuals with available Lp(a) concentrations in our dataset (n = 173,878) had Lp(a) concentrations <3.9 and >189 nmol/L, respectively. Measurements above the analytical range were reanalyzed with serial dilutions as described in: https://biobank.ndph.ox.ac.uk/showcase/showcase/docs/serum_biochemistry.pdf.

### 2.3 Variant typing in GCKD, KORA F3 and KORA F4

In GCKD, rs41272110 was represented directly on the genotyping microarray. In KORA F3 and F4 genotypes were retrieved from Haplo-type Reference Consortium-imputed data [34] using the Imputation Server [35] (imputation quality for KORA F3: R^2^ = 0.96953 and KORA F4: R^2^ = 0.95874). Genotyping arrays were Illumina Omni2.5Exome array for GCKD, Illumina Omni2.5 and Illumina Omni Express for KORA F3 and Affymetrix Axiom for KORA F4 with call rates >98%. KIV-2 4925G>A carrier status had been determined earlier by amplification-refractory TaqMan PCR (described in [10,14]).

### 2.4 Statistical analysis

Because of the technical limitations of genotyping a variant within a hypervariable copy number array, presence of KIV-2 4925G>A on multiple KIV-2 copies of the same chromosome cannot be distinguished from being present on different chromosomes [10,13]. Therefore, KIV-2 4925G>A is reported as positive or negative carrier status [10,13]. Where required, 4925G>A carriers were coded as heterozygous, as done previously [10,13]. LD values (R^2^, Lewontin’s D’) were tested using the R package *genetics* [36]. In each study population the association between variants and Lp(a) concentrations was tested by multiple quantile regression, estimating the conditional median of Lp(a) to account for the skewed distribution, using the R package *quantreg* [37]. The models were adjusted for age and sex (regression model 1) as well as the apo(a) isoform (regression model 2). The smaller isoform is most often the dominant isoform [38] and commonly used for classification in epidemiological studies [39]. Thus in heterozygous individuals showing two isoforms in Western blot the smaller isoform of the two was used. In true homozygous individuals and individuals expressing only one isoform the only isoform present on the Western Blot was used. The apo (a) isoform reflects the expressed KIV-2 repeat number on continuous scale. The smaller apo(a) isoform is also used for grouping. In GCKD, both models were further adjusted for the estimated glomerular filtration rate (eGFR), calculated as stated in [40]. Where required, rs41272110 was additionally adjusted for 4925G>A and *vice versa* (termed as joint model in this manuscript). Interaction between the variants was tested using the quantile regression model adjusted for the smaller apo(a) isoform (regression model 2). Sensitivity analysis to exclude a role of hidden population substructures was performed on restricted datasets of GCKD (n = 4575) and KORA F3 (n = 2782) excluding known first- and second-degree relationships and with further adjustment for the first 10 (GCKD) and 8 (KORA F3) principal components. Principal component calculation condenses unobserved factors that contribute to variance into a smaller set of computed variables [41]. They were calculated from the genome wide genotype matrix to account and adjust for population stratification. Sensitivity analysis used quantile regression model 2. Differences in medians were assessed using Kruskal-Wallis and Dunn’s test. Random-effect meta-analysis was performed using the R package *metafor* [42]. All analysis on GCKD, KORA F3 and KORA F4 were done in R version 4.0.1.

### 2.5 Analysis of UK biobank

UK Biobank data were restricted to self-reported European ancestry (British, Irish or any other white ethnic background). Rs41272110 and 4925G>A data and Lp(a) concentrations were available for n = 173,878. The impact of rs41272110 and 4925G>A on Lp(a) concentrations and the interaction between both variants was investigated using quantile regression. As no apo(a) isoform data are available in UKB, only regression model 1 was calculated. The 4925G>A variant was retrieved from the UKB whole exome sequencing data using the strategy described by Ebbert et al. [43] with slight modifications (Supplementary Methods).

Hazard ratio (HR) for CAD was estimated using a Cox model as a function of the carrier status of the two SNPs, with participant age as timescale and additionally adjusted for sex. SNPs were considered both independently from each other and in a joint model (i.e. one variant adjusted for the other). CAD was defined by the ICD-10 codes I21 to I25 [44]. Variant and CAD outcome data were available for 186,088 individuals. The visual assessment of Schoenfeld residuals to check proportional hazard assumptions showed some deviation over time for sex and 4925G>A carrier status. However, no major difference was found when the Cox proportional hazard model was performed in patients stratified by age (cut-off: median age at recruitment, <58 and ≥58 years).

In UKB sensitivity analysis was performed on a restricted dataset without genetic kinship (first-, second- or third-degree relationships). Each model was adjusted for the first 30 principal components. Quantile regression was performed on 120,228 individuals with available Lp(a) measurements and survival analysis on 128,672 individuals including 8,922 CAD cases.

Further information on the analysis of UKB is available in the Supplementary Methods. Analyses in UKB were performed using R version 3.6.3.

## 3 Results

### 3.1 Both rs4272110 and 4925G>A decrease Lp(a) in single SNP analysis

Rs41272110 is a long-known missense variant in *LPA* with an often assumed causal impact on Lp(a) concentrations [19–21,23–25]. The current human genome annotation system assigns it to p.Thr1399Pro, but it is better known with the non-standard designation Thr3888Pro (based on an older reference protein for apo(a) [UniProt [45] P08519]).

Meta-analysis on the overall population using quantile regression adjusted for age and sex showed no effect of rs41272110 on Lp(a) concentration (β = –0.06, *p* = 0.879; regression model 1; Table 2, study-wise results shown in Supplementary Table II). However, stratification by isoform into LMW (at least one isoform with 10–22 KIV repeats) and HMW (only isoforms with >22 KIV repeats) carriers, revealed a highly significant decreasing effect of rs41272110 in LMW carriers (β = –24.28, *p* = 1.05e-27), which was not seen in HMW carriers, where it presented rather a small but significant Lp(a)-increasing effect (β = +1.69, *p* = 6.13e-07). This suggested a confounding effect of the isoform background.

This assumption was confirmed by adjusting the regression model by the smaller apo(a) isoform (regression model 2, Table 2, study-wise results shown in Supplementary Table II). This revealed an association of rs41272110 with reduced Lp(a) in the overall population (β = –12.34, *p* = 3.93e-159), as well as in the LMW group (β = –23.90, *p* = 6.37e-22). Conversely, in the HMW group the effect remained small, but became negative (β = –1.06, *p* = 3.22e-06). These observations suggest that isoform size modifies the association signal of rs41272110 and strongly reminds the association patterns that we have recently described for Lp(a) and the KIV-2 variant 4925G>A [10].

For KIV-2 4925G>A, we observed an association with significantly decreased Lp(a) concentrations (model 1: β = –2.28, *p* = 4.83e-06; model 2: β = –20.52, *p* = 7.54e-77) and the effect was markedly stronger in LMW than in HMW apo(a) isoforms (Table 2, study-wise results shown in Supplementary Table II), which is in line with previous findings [10,13]. Sensitivity analysis on the restricted datasets without related individuals and adjusted for potential population stratification did not affect the beta estimates (Supplementary Table III).

### 3.2 The effect of rs41272110 on Lp(a) is modified by KIV-2 4925G>A

A highly significant SNP-SNP interaction was detected between rs41272110 and 4925G>A in all populations (GCKD: *p* = 3.62e-05, KORA F3: *p* = 1.26e-05, KORA F4: *p* = 3.03e-04). Additionally, both variants were observed in the same isoform range (Supplementary Figure I) and show a high LD (R^2^ = 0.836–0.858; D’ = 0.984–0.985, Table 1). The high D’ indicates that little recombination is observed between the two loci. The two loci, respectively SNPs alleles are often inherited together. D’ is a suitable measure for LD when the MAFs of the two variants differ, as this prevents R^2^ from reaching the maximal value of 1.

When analyzed individually, both variants were associated with a pronounced Lp(a) decrease (Table 2, Supplementary Table II). However, adjusting rs41272110 for 4925G>A (joint model) surprisingly inverted the effect of rs41272110, which became pronouncedly positive in the overall population (model 1: β = +9.40, *p* = 4.07e-10; model 2: β = +4.43, *p* = 2.31e-12), as well as in the LMW/HMW subgroups (Table 2, Supplementary Table IV). Vice versa, adjusting 4925G>A for rs41272110 strengthened the association of 4925G>A with lower Lp(a) (Table 2, Supplementary Table IV).

Due to the higher MAF of rs41272110, 14.9% of rs41272110 carriers do not carry KIV-2 4925G>A (Table 3), despite of the strong LD. Therefore, we stratified the populations in four genotype combinations. These were individuals carrying only rs41272110, individuals carrying only 4925G>A, individuals carrying both variants (double carriers) and individuals carrying neither of both (wild type; reference group in the regression model).

**Fig. 1:**
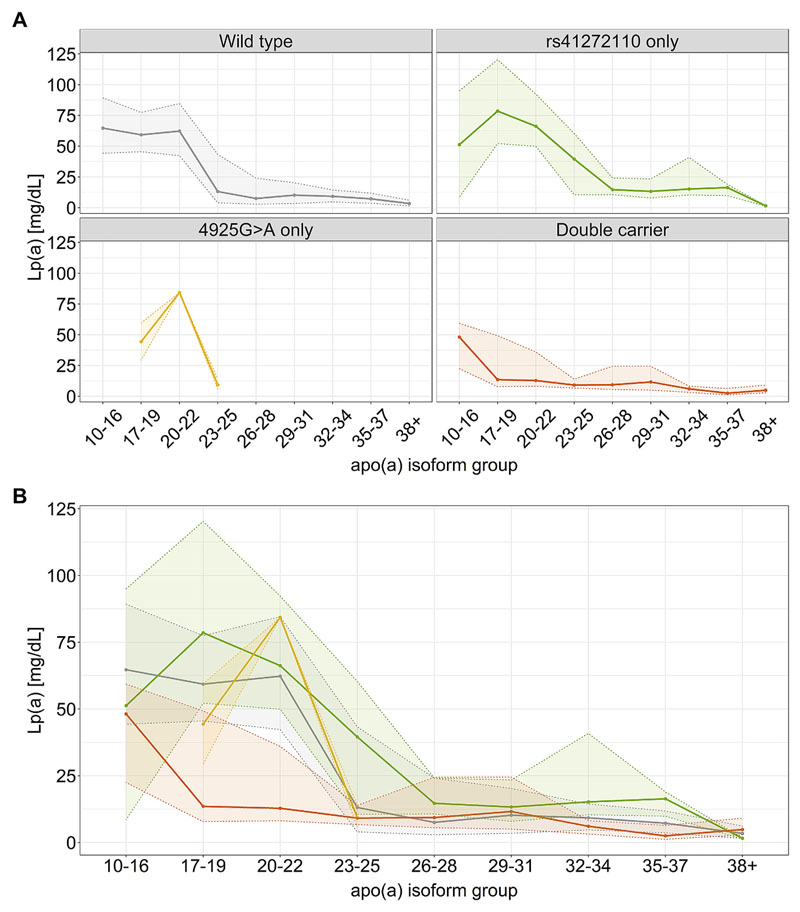
Median Lp(a) concentration of the genotype combinations over the whole isoform range in the GCKD population. (A) Single distributions of all genotype combinations; (B) superimposed Lp(a) distributions of all genotype combinations. Compared to wild type Lp(a) concentrations are reduced in double carriers but increased in individuals carrying rs41272110 only. Solid line represents the median Lp(a) concentration. Ribbon represents the interquartile range. GCKD is representative for all study populations used, KORA F3 and F4 are shown in Supplementary Figures VI and VII. This figure shows the isoform distribution of the individuals with the given SNP combination. The isoform distribution of the single variants are shown in Supplementary Fig. I.

We observed a highly significant difference in median Lp(a) concentrations between rs41272110-only and double carriers (25.57–39.26 mg/dL difference depending on the study, *p* = 2.91e-20 to 1.71e-23), whereas no difference was seen between 4925G>A-only and double carriers (*p* = 0.58–0.97; 1.66–11.52 mg/dL difference). Rs41272110-only carriers tended to have higher Lp(a) medians than wild types, while double carriers show a marked decrease in Lp(a) (medians are shown in Supplementary Table V and respective box plots are shown in Supplementary Figure II). These effects were observed over the whole isoform range (Fig. 1, Supplementary Figures III-V) and were most pronounced in isoforms with 17–25 KIV, which corresponds to the isoform range with the highest carrier frequency of 4925G>A [10] (Supplementary Figures VI-VIII).

These observations were confirmed by performing regression models 1 and 2 using the genotype combinations (Table 3, Supplementary Table VI). In all analyses carriers of rs41272110 showed significantly increased Lp(a) concentrations, while 4925G>A-only and double carriers showed similar Lp(a)-lowering effects in population-wide analyses, but 4925G>A-only status showed stronger Lp(a)-lowering in the LMW group, which is likely driven by the previously described strong impact of 4925G>A in this group [10,13].

### 3.3 The effect on coronary artery disease in UK biobank depends on the rs41272110-4925G>A genotype combination

Quantile regression analysis on Lp(a) in the UKB replicated the findings from GCKD, KORA F3 and KORA F4 (Supplementary Tables VII and VIII; only model 1 can be given due to the lack of apo(a) isoform data in UKB) and the SNP interaction test was highly significant in the UKB as well (*p* = 2.79e-56).

**Fig. 2:**
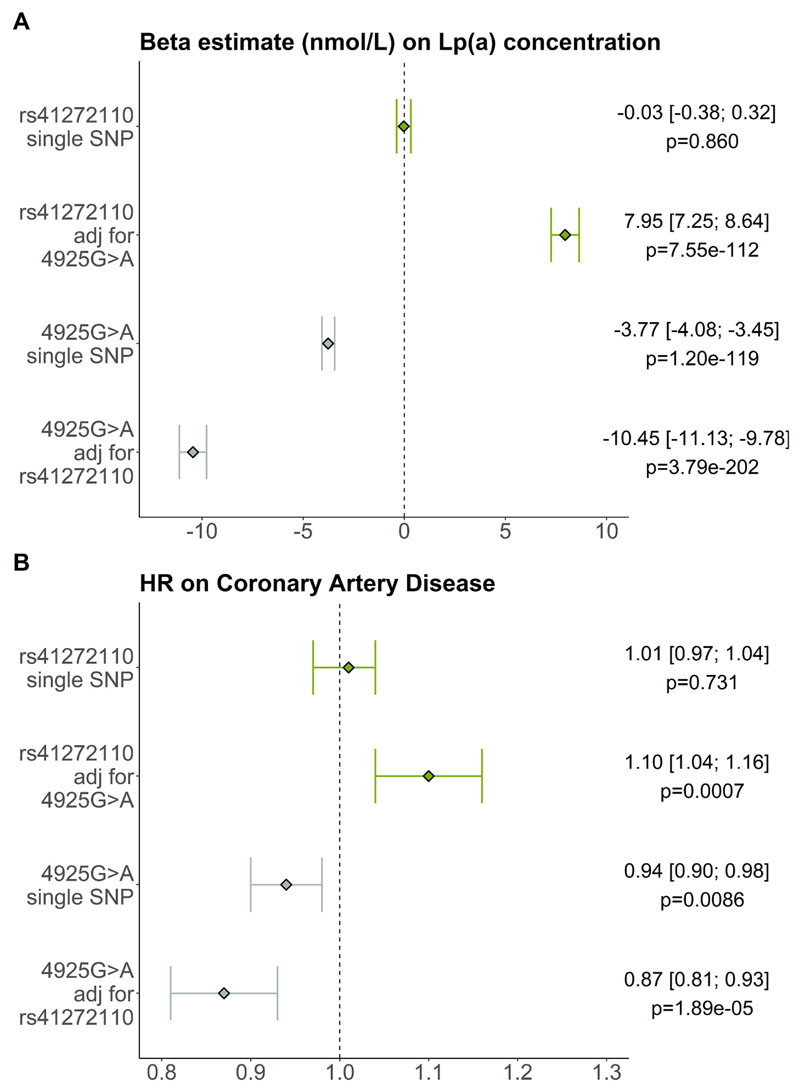
Effect of the variants on Lp(a) concentration and on CAD risk. (A) Effects of the variants on Lp(a) concentration in nmol/L in the UK Biobank (n = 173,878). Quantile regression models were adjusted for age and sex. (B) Hazard ratios (HR) of rs41272110 and 4925G>A in UK Biobank (n = 186,088). Rs41272110 carriers (n = 50,228) show no reduced risk for CAD (total CAD events = 13,335; 3,612 CAD events in rs41272110-carriers). KIV-2 4925G>A carriers (n = 32,627) show significantly reduced risk for CAD (total CAD events = 13,335; 2,229 CAD events in 4925G>A carriers). In a joint model (one variant adjusted for the other), hazard ratios show increased risk for CAD events in rs41272110 carriers (n = 50,228) and reduced risk for CAD in 4925G>A carriers (n = 32,627). All models are adjusted for sex and take participant age as timescale. adj: adjusted.

In Cox regression analysis using participant age as timescale, 4925G>A (2,229 cases in 32,627 carriers) showed a significantly reduced CAD risk (HR = 0.94 [0.90; 0.98], *p* = 0.0086; Fig. 2), while rs41272110 alone (3,612 cases in 50,228 carriers) did not show an association with CAD (HR = 1.01 [0.97; 1.04], *p* = 0.731; Fig. 2).

On the contrary, adjustment of rs41272110 for 4925G>A reveals a significant increase of CAD risk for rs41272110 (HR = 1.10 [1.04; 1.16], *p* = 0.0007; Fig. 2). This is in line with the significant Lp(a)-increase that is observed in rs41272110-only individuals. *Vice versa,* the protective effect of 4925G>A on CAD was strengthened by adjusting 4925G>A for rs41272110 (HR = 0.87 [0.81; 0.93], *p* = 1.89e-05, Fig. 2), which is consistent with the even stronger Lp(a) reduction that has been observed in this model (Supplementary Table VII). After adjustment for Lp(a) concentration, the association between the variants and CAD risk was strongly alleviated and partially abolished, confirming the expected imperative role of the Lp(a) concentrations as shown before [13,46,47].

Sensitivity analyses on the restricted dataset further adjusted for 30 principal components did change the beta estimates (Supplementary Table VIII) and the CAD risk figures only marginally (Supplementary Figure XI) and the SNP-SNP interaction remained highly significant (*p* = 4.17e-17).

## 4 Discussion

Our study contains several complexities that require extensive discussion. Rs41272110 leads to an amino acid substitution from threonine to proline (p.Thr1399Pro/Thr3888Pro) that may affect the Lp (a) assembly and its characteristics [18]. Prins et al. described the variant first and found an association with reduced risk of symptomatic atherosclerosis, despite non-elevated Lp(a) [18]. Several subsequent studies investigated the association between this missense variant and Lp(a) concentrations but found inconsistent results, reporting associations with both decreased and increased Lp(a) concentrations. The observations of these studies are summarized in Supplementary Table I [18–21,23–25].

We coincidentally observed that rs41272110 and KIV-2 4925G>A show similar MAF and similar apo(a) isoform distribution patterns and hypothesized that the two variants might be in partial LD. Subsequent in-depth analyses revealed that the true effect of isolated rs41272110 carrier status on Lp(a) and CAD risk is indeed modified by an interaction with KIV-2 4925G>A, as well as by an association with specific apo(a) isoform ranges. The size and direction of the association of rs41272110 with Lp(a) depend strongly on whether the interaction between the variants is considered (graphical abstract). In an unadjusted model including only rs41272110, as done in some previous studies [18,23, 24], no effect of rs41272110 on Lp(a) is detectable. Conversely, additional adjustment of the regression models for apo(a) isoforms results in a marked association of rs4127210 with *lower* Lp(a) concentrations and *lower* CAD risk. This is, however, in turn caused by a partial LD of rs41272110 with the strongly Lp(a)-lowering splice site variant KIV-2 4925G>A. Accounting for this finally reveals that isolated rs41272110 is actually associated with markedly *higher* Lp(a) concentrations and *higher* CAD risk.

At population scale, this effect has been missed before because of the partial LD with the strongly Lp(a)-lowering effect of 4925G>A. About 85% of the rs41272110 carriers also carry 4925G>A, which leads to a net Lp(a)-lowering effect of rs41272110 on population level. However, this perceived lowering effect of rs41272110 is not applicable to indeed 15% of the SNP carriers, which corresponds to sizeable 4% of the population. Thus, a conspicuous share of the population would be wrongly classified if only rs41272110 is considered. This highlights how the effect of a single SNP determined by population-wide association studies may not necessarily be transferrable to single individuals with different genetic background. Differing proportions of single vs. double carriers might also explain the inconsistent results of previous investigations on rs41272110 [18–21,24,25]. Indeed, it has been repeatedly reported that LD patterns in *LPA* strongly determine and/or modify the net effect of the involved SNPs [14,26,48–50].

Mechanistically, one would assume an additive effect of both variants on Lp(a) concentrations. This would lead to a lower decrease in double carriers compared to individuals presenting only 4925G>A. However, we observe that double carriers show a similar Lp(a)-decreasing effect as carriers of 4925G>A only. This observation indicates that rs41272110 and 4925G>A are located on the same gene allele, as supported also by the observed LD. Since 4925G>A induces a partial splicing defect that decreases mRNA production [10], it offsets any possible effect of the amino acid change caused by rs41272110.

Finally, we show in UKB, a large-scale prospective study, that the LD between rs41272110 and 4925G>A also affects CAD risk assessment significantly. Since isoform-adjustment is not possible in UKB, rs41272110 shows no effect on CAD at first glance. However, when adjusted for 4925G>A, rs41272110 is associated with markedly higher Lp(a) concentrations and confers a significant, 10% higher CAD risk. Of note, individuals with rs41272110 but not 4925G>A represented 9.8% of the UKB study population, compared to 4% in our studies, which may be due to different variant typing of 4925G>A (exome sequencing versus amplification-refractory TaqMan PCR) but also due to previously shown inter-European differences in the Lp(a) trait and in carriers of 4925G>A [10,32].

Our findings may guide biochemical characterization of this long-known missense variant into a new direction. An Lp(a)-increasing effect of an amino acid exchange would be very intriguing and mechanistic data would be interesting. However, our results do not yet indicate that the Lp(a)-raising effect observed in Thr3888Pro-only carriers is caused by the amino acid change itself. It could also be caused by an LD with another yet unidentified functional SNP that is found on the same haplotype as Thr3888Pro-only. Biochemical studies are warranted to elucidate this question.

Overall, our findings reinforce the complex genetic architecture of plasma Lp(a) and the intricate impact of *LPA* SNPs on CAD [10,11,13,14, 17]. *LPA* SNPs are important contributors to genetic risk scores (GRS) for CAD [47,51,52] and Lp(a) [47,51–54]. These GRS have been shown to explain up to 70% of Lp(a) variance, resemble directly measured Lp(a) concentrations reasonably and are efficient screening tools to identify hyper-Lp(a)emic patients using available genetic data [47,53,54]. Inclusion of non-additive effects, SNP interactions, haplotypes and SNPs that define subgroups within more frequent haplotypes may refine Lp(a) and CAD GRS further [11] and improve genetic classification of hidden patient subpopulations. This may further pave the way of GRS to clinical application, as genomic data becomes increasingly available.

### 4.1 Strengths and limitations

Using three large datasets including apo(a) isoform sizes and SNPs data from with >10,000 individuals, as well as UK Biobank data of ≈174,000–186,000 individuals, we identified a SNP-SNP interaction between rs41272110 (Thr3888Pro) and KIV-2 4925G>A and showed an additional modifying role of the apo(a) background isoform. We show that this strongly affects the perceived effects of rs41272110 (Thr3888Pro) on Lp(a) and CAD risk.

We acknowledge that this study focuses on two specific variants. Indeed, our observations highlight the need of scaling such analyses up to locus- or genome-wide approaches. Unfortunately, since 4925G>A is located in the KIV-2 repeat, only the carrier status of the variant was available [10,13], precluding formal haplotype analysis with phased data. Upcoming advanced phasing procedures capable of phasing also KIV-2 variants [11] may hopefully allow to refine this in future. We also noted a rather high heterogeneity estimate (I^2^) in our meta-analysis although the relative difference in effect estimates is moderate. This can be explained by study-wise high effect estimates and high number of cases. Lp(a) measurements in GCKD/KORA F3/KORA F4 and in UK Biobank were performed with two different assays. Concentrations are given in mg/dL for the former and in nmol/L for the latter. An isoform-sensitivity of both assay cannot entirely be excluded. However, the high effect estimates of both variants cannot be explained by a possible bias generated by the assay. Furthermore, both variants occur predominantly in the same isoform range and would be affected equally. Importantly, our results are limited to white European ancestry. We hope that our findings will be followed-up by studies in more diverse and in underrepresented populations.

### 4.2 Conclusion

In conclusion, we reveal a hitherto undetected Lp(a)-increasing effect of rs41272110 (Thr3888Pro), which, at population-scale, is missed due to an interaction with KIV-2 4925G>A and an association with isoforms. This reveals a novel risk variant for CAD present in a sizeable yet previously missed subgroup of the population and emphasizes how in the *LPA* gene unrecognized interactions might mislead the interpretation of genetic risk profiles.

## Supplementary Material

Supplementary data to this article can be found online at https://doi.org/10.1016/j.atherosclerosis.2022.04.023.

Supplementary File

## Figures and Tables

**Table 1:** Characteristics of the study populations.

	GCKD	KORA F3	KORA F4	UKB
n	4,575	2,939	2,891	186,088
Age, y ^a^	63 (53, 70)	57 (46, 67)	56 (44, 67)	58 (50, 63) ^c^
Female sex ^b^	1,786 (39.8)	1,496 (51.8)	1,467 (51.9)	102,627 (55.2%)
Lp(a) ^a^	11.49 mg/dL (4.86, 34.51)	11.04 mg/dL (4.85, 29.59)	11.70 mg/dL (5.17, 31.03)	18.6 nmol/L (7.39, 72.02)
eGFR, ml/min/1.73 m^2 a^	46 (37, 58)	85 (72, 95)	85 (73, 96)	NA
Total cholesterol, mg/dL ^a^	207 (177, 239)	216 (191, 243)	214 (188, 240)	220 (192, 251)
HDL-C, mg/dL ^a^	48 (39, 61)	56 (46, 68)	54 (45, 65)	55 (46, 65)
LDL-C, mg/dL ^a^	114 (89, 143)	126 (105, 148)	134 (111, 158)	137 (115, 160)
Triglycerides, mg/dL ^a^,	169 (118, 239)	135 (88, 200)	105 (72, 151)	131 (93, 190)
Type 2 diabetes mellitus ^b^	1,631 (35.7)	215 (7.3)	200 (6.9)	8,060 (4.5)
4925G>A carrier ^b^	926 (20.2)	687 (23.4)	642 (22.2)	32,627 (17.5)
4925G>A MAF, %	10.7	12.5	11.8	9.2
rs41272110 carrier ^b^	1,151 (25.2)	795 (27.1)	753 (26.0)	50,228 (27.0)
rs41272110 MAF, %	13.5	14.4	13.9	13.5
Interaction between rs41272110 and 4925G>A	p = 3.62e-05	p = 1.26e-05	p = 3.03e-04	p = 2.79e-56
LD between rs41272110 and 4925G>A	R^2^ = 0.836,	R^2^ = 0.872,	R^2^ = 0.858,	R^2^ = 0.731,
	D’ = 0.985	D’ = 0.984	D’ = 0.985	D’ = 0.975

UK Biobank cohort is restricted to self-reported European ancestry (British, Irish or any other white ethnic background), with available genotype data for rs41272110 and exome data for 4925G>A. Total cholesterol, HDL-C and LDL-C are converted from mmol/L to mg/dL by multiplying using conversion factor 38.67 and triglycerides using factor 88.57. NA: not available.

a Values are provided as median (interquartile range).

b Values are provided as number of patients (%).

c Age at recruitment.

**Table 2:** Meta-analysis of quantile regression analysis of rs41272110 and 4925G>A on Lp(a) concentration in GCKD, KORA F3 and KORA F4, modelling both SNPs independently (upper part) and in a joint model (lower part). Effect given in mg/dL. In GCKD both models were further adjusted for eGFR. I^2^ explains the estimated fraction of variance due to heterogeneity between the three datasets.

			Model 1: adjusted for age and sex				Model 2: adjusted for age, sex and smaller apo(a) isoform			
Group	Variant	Carrier [n]	β	95% CI	*p*-value	I^2^ [%]	β	95% CI	*p*-value	I^2^ [%]
Overall population	rs41272110	2,699	−0.06	−0.79, 0.68	0.879	62.0	−12.34	−13.22, −11.46	3.93E-159	86.7
	4925G>A	2,255	−2.28	−3.25, −1.30	4.83E-06	64.3	−20.52	−22.67, −18.37	7.54E-77	81.0
LMW	rs41272110	896	−24.28	−28.59, −19.97	1.05E-27	42.9	−23.90	−28.72, −19.08	6.37E-22	56.0
	4925G>A	717	−43.96	−49.76, −38.15	1.08E-31	86.7	−42.82	−49.89, −35.75	1.74E-25	90.2
HMW	rs41272110	1,802	+1.69	1.03, 2.36	6.13E-07	70.3	−1.06	−1.51, −0.62	3.22E-06	1.9
	4925G>A	1,537	+0.63	−0.42, 1.67	0.240	81.7	−3.57	−4.59, − 2.55	6.83E-12	59.4
**Joint model (one variant adjusted for the other)**										
Overall population	rs41272110	2,699	+9.40	6.45, 12.34	4.07E-10	0	+4.43	3.20, 5.67	2.31E-12	0
	4925G>A	2,255	−11.95	−14.95, − 8.95	6.41E-15	0	−25.55	−27.64, − 23.46	1.37E-123	39.1
LMW	rs41272110	896	+5.34	0.04, 10.64	0.0482	69.0	+7.23	2.84, 11.61	0.00125	41.0
	4925G>A	717	−48.85	−57.43, − 40.27	3.09E-28	82.5	−49.71	−57.95, − 41.46	2.07E-31	76.4
HMW	rs41272110	1,802	+6.28	4.04, 8.52	3.83E-08	74.8	+5.10	3.72, 6.47	3.85E-13	50.5
	4925G>A	1,537	−5.68	−8.02, − 3.34	1.99E-06	74.6	−8.70	−10.73, − 6.67	4.90E-17	67.4

**Table 3:** Meta-analysis of quantile regression model on the genotype combinations in GCKD, KORA F3 and KORA F4. Populations were stratified into four genotype combination categories: individuals carrying only rs41272110, individuals carrying only 4925G>A, individuals carrying both variants (double carriers) and individuals carrying neither of both (defined here as wild type). Wild type individuals represents the reference group in the regression model. Effect given in mg/dL. In GCKD both models were further adjusted for eGFR. I^2^ explains the estimated fraction of variance due to heterogeneity between the three datasets.

			Model 1: adjusted for age and sex				Model 2: adjusted for age, sex and smaller isoform			
Group	Variant	Carrier [n]	β	95% CI	*p*-value	I^2^ [%]	β	95% CI	*p*-value	I^2^ [%]
All	Wild type	7,678	reference							
	rs41272110 only	472	+28.96	19.89, 38.02	4.02E-10	97.5	+14.30	8.57, 20.03	1.01E-06	94.5
	4925G>A only	28	+1.86	−7.05, 10.78	0.682	61.1	−15.93	−23.99, −7.88	0.0001	58.4
	Double carriers	2,227	−1.54	−2.56, −0.53	2.82E-03	50.6	−19.79	−21.90, −17.69	3.20E-74	88.7
LMW	Wild type	1,618	reference							
	rs41272110 only	186	+11.91	3.86, 19.97	3.76E-03	76.7	+14.06	6.20, 21.91	0.0005	74.1
	4925G>A only	7	−15.35	−47.80, 17.10	0.354	64.9	−15.58	−47.84, 16.69	0.344	62.8
	Double carriers	710	−42.79	−48.60, −36.98	1.31E-46	85.6	−41.21	−48.00, −34.42	2.22E-32	88.8
HMW	Wild type	6,057	reference							
	rs41272110 only	286	+12.70	6.82, 18.59	2.35E-05	93.2	+10.26	5.79, 14.73	7.01E-06	93.3
	4925G>A only	21	+3.23	−0.29, 6.75	0.0725	23.6	−1.92	−5.97, 2.12	0.351	0
	Double carriers	1,516	−41.11	−47.92, −34.29	5.36E-32	74.0	−2.97	−3.76, − 2.19	1.32E-13	39.8

## References

[1] Kronenberg F, Utermann G (2013). Lipoprotein(a): resurrected by genetics. J Intern Med.

[2] Laschkolnig A, Kollerits B, Lamina C, Meisinger C, Rantner B, Stadler M, Peters A, Koenig W, Stöckl A, Dahnhardt D, Böger CA (2014). Lipoprotein (a) concentrations, apolipoprotein (a) phenotypes, and peripheral arterial disease in three independent cohorts. Cardiovasc Res.

[3] Nordestgaard BG, Langsted A (2016). Lipoprotein(a) as a cause of cardiovascular disease: insights from epidemiology, genetics, and biology. J Lipid Res.

[4] Kamstrup PR, Nordestgaard BG (2016). Elevated lipoprotein(a) levels, LPA risk genotypes, and increased risk of heart failure in the general population. JACC Hear Fail.

[5] Langsted A, Kamstrup PR, Nordestgaard BG (2019). High lipoprotein(a) and high risk of mortality. Eur Heart J.

[6] Larsson SC, Gill D, Mason AM, Jiang T, Böck M, Butterworth AS, Burgess S (2020). Lipoprotein(a) in alzheimer, atherosclerotic, cerebrovascular, thrombotic, and valvular disease. Circulation.

[7] Arnold M, Schweizer J, Nakas CT, Schütz V, Westphal LP, Inauen C, Pokorny T, Luft A, Leichtle A, Arnold M, Bicvic A (2021). Lipoprotein(a) is associated with large artery atherosclerosis stroke aetiology and stroke recurrence among patients below the age of 60 years: results from the BIOSIGNAL study. Eur Heart J.

[8] Cohen JC, Chiesa G, Hobbs HH (1993). Sequence polymorphisms in the apolipoprotein (a) gene. Evidence for dissociation between apolipoprotein(a) size and plasma lipoprotein(a) levels. J Clin Invest.

[9] Perombelon YFN, Soutar AK, Knight BL (1994). Variation in lipoprotein(a) concentration associated with different apolipoprotein(a) alleles. J Clin Invest.

[10] Coassin S, Erhart G, Weissensteiner H, Eca Guimarães de Araújo M, Lamina C, Schönherr S, Forer L, Haun M, Losso JL, Köttgen A, Schmidt K (2017). A novel but frequent variant in LPA KIV-2 is associated with a pronounced Lp(a) and cardiovascular risk reduction. Eur Heart J.

[11] Mukamel RE, Handsaker RE, Sherman MA, Barton AR, Zheng Y, McCarroll SA, Loh P-R (2021). Protein-coding repeat polymorphisms strongly shape diverse human phenotypes. Science.

[12] Said MA, Yeung MW, van de Vegte YJ, Benjamins JW, Dullaart RPF, Ruotsalainen S, Ripatti S, Natarajan P, Juarez-Orozco LE, Verweij N, van der Harst P (2021). Genome-wide association study and identification of a protective missense variant on lipoprotein(a) concentration. Arterioscler Thromb Vasc Biol.

[13] Schachtl-Riessang JF, Kheirkhah A, Grüneis R, Di Maio S, Schoenherr S, Streiter G, Losso JL, Paulweber B, Eckardt K-U, Kottgen A, Lamina C (2021). Frequent LPA KIV-2 variants lower lipoprotein(a) concentrations and protect against coronary artery disease. J Am Coll Cardiol.

[14] Di Maio S, Grüneis R, Streiter G, Lamina C, Maglione M, Schoenherr S, Oöfner D, Thorand B, Peters A, Eckardt K-U, Koöttgen A (2020). Investigation of a nonsense mutation located in the complex KIV-2 copy number variation region of apolipoprotein(a) in 10,910 individuals. Genome Med.

[15] Kraft HG, Windegger M, Menzel HJ, Utermann G (1998). Significant impact of the +93 C/T polymorphism in the apolipoprotein(a) gene on Lp(a) concentrations in Africans but not in Caucasians: confounding effect of linkage disequilibrium. Hum Mol Genet.

[16] Trinder M, Brunham LR (2020). Polygenic modulation of lipoprotein(a)-associated cardiovascular risk, Preprint. MedRxiv.

[17] Zeng L, Moser S, Mirza-Schreiber N, Lamina C, Coassin S, Nelson CP, Annilo T, Franzén O, Kleber ME, Mack S, Andlauer TFM (2022). Cis-epistasis at the LPA locus and risk of cardiovascular diseases. Cardiovasc Res.

[18] Prins J, Leus FR, Van Der Hoek YY, Kastelein JJP, Bouma BN, Van Rijn HJM (1997). The identification and significance of a Thr→Pro polymorphism in kringle IV type 8 of apolipoprotein(a). Thromb Haemostasis.

[19] Chretien JP, Coresh J, Berthier-Schaad Y, Kao WHL, Fink NE, Klag MJ, Marcovina SM, Giaculli F, Smith MW (2006). Three single-nucleotide polymorphisms in LPA account for most of the increase in lipoprotein(a) level elevation in African Americans compared with European Americans. J Med Genet.

[20] Ogorelkova M (2001). Single nucleotide polymorphisms in exons of the apo(a) kringles IV types 6 to 10 domain affect Lp(a) plasma concentrations and have different patterns in Africans and Caucasians. Hum Mol Genet.

[21] Prins J, Leus FR, Bouma BN, van Rijn HJ (1999). The identification of polymorphisms in the coding region of the apolipoprotein (a) gene-association with earlier identified polymorphic sites and influence on the lipoprotein (a) concentration. Thromb Haemostasis.

[22] Hirschfeldova K, Lipovska D, Skrha P, Ceska R (2009). The apo(a) gene (TTTTA)n promoter polymorphism and its association with variability in exons of the kringle IV types 8 to 10. Clin Chim Acta.

[23] Yahya R, Berk K, Verhoeven A, Bos S, van der Zee L, Touw J, Erhart G, Kronenberg F, Timman R, Sijbrands E, Roeters van Lennep J (2019). Statin treatment increases lipoprotein(a) levels in subjects with low molecular weight apolipoprotein(a) phenotype. Atherosclerosis.

[24] Deo RC, Wilson JG, Xing C, Lawson K, Kao WHL, Reich D, Tandon A, Akylbekova E, Patterson N, Mosley TH, Boerwinkle E (2011). Single-Nucleotide polymorphisms in LPA explain most of the ancestry-specific variation in lp(a) levels in african Americans. PLoS One.

[25] Lee S-R, Prasad A, Choi Y-S, Xing C, Clopton P, Witztum JL, Tsimikas S (2017). LPA gene, ethnicity, and cardiovascular events. Circulation.

[26] Coassin S, Hermann-Kleiter N, Haun M, Wahl S, Wilson R, Paulweber B, Kunze S, Meitinger T, Strauch K, Peters A, Waldenberger M (2020). A genome-wide analysis of DNA methylation identifies a novel association signal for Lp(a) concentrations in the LPA promoter. PLoS One.

[27] Titze S, Schmid M, Koöttgen A, Busch M, Floege J, Wanner C, Kronenberg F, Eckardt K-U, Eckardt K-U, Titze S, Prokosch H-U (2015). For the G. study investigators, Disease burden and risk profile in referred patients with moderate chronic kidney disease: composition of the German Chronic Kidney Disease (GCKD) cohort. Nephrol Dial Transplant.

[28] Eckardt K-U, Baörthlein B, Baid-Agrawal S, Beck A, Busch M, Eitner F, Ekici AB, Floege J, Gefeller O, Haller H, Hilge R (2012). The German Chronic Kidney Disease (GCKD) study: design and methods. Nephrol Dial Transplant.

[29] Wichmann H-E, Gieger C, Illig T (2005). For the Group MS KORA-gen - resource for population genetics, controls and a broad spectrum of disease phenotypes. Gesundheitswesen.

[30] Bycroft C, Freeman C, Petkova D, Band G, Elliott LT, Sharp K, Motyer A, Vukcevic D, Delaneau O, O’Connell J, Cortes A (2018). The UK Biobank resource with deep phenotyping and genomic data. Nature.

[31] Dieplinger H, Gruber G, Krasznai K, Reschauer S, Seidel C, Burns G, Müller HJ, Cséaszaér A, Vogel W, Robenek H (1995). Kringle 4 of human apolipoprotein[a] shares a linear antigenic site with human catalase. J Lipid Res.

[32] Erhart G, Lamina C, Lehtimaöki T, Marques-Vidal P, Kaöhoönen M, Vollenweider P, Raitakari OT, Waeber G, Thorand B, Strauch K, Gieger C (2018). Genetic factors explain a major fraction of the 50% lower lipoprotein(a) concentrations in Finns. Arterioscler Thromb Vasc Biol.

[33] Kronenberg F, Kuen E, Ritz E, Junker R, Konig P, Kraatz G, Lhotta K, Mann JF, Muller GA, Neyer U, Riegel W (2000). Lipoprotein(a) serum concentrations and apolipoprotein(a) phenotypes in mild and moderate renal failure. J Am Soc Nephrol.

[34] McCarthy S, Das S, Kretzschmar W, Delaneau O, Wood AR, Teumer A, Kang HM, Fuchsberger C, Danecek P, Sharp K, Luo Y (2016). A reference panel of 64,976 haplotypes for genotype imputation. Nat Genet.

[35] Das S, Forer L, Schöonherr S, Sidore C, Locke AE, Kwong A, Vrieze SI, Chew EY, Levy S, McGue M, Schlessinger D (2016). Next-generation genotype imputation service and methods. Nat Genet.

[36] Warnes Gregory, Gorjanc Gregor, Leisch Friedrich, Man Michael (2019). genetics: population Genetics. R package version 1.3.8.1.2.

[37] Koenker R (2020). Quantreg: Quantile Regression.

[38] Gaubatz JW, Ghanem KI, Guevara J, Nava ML, Patsch W, Morrisett JD (1990). Polymorphic forms of human apolipoprotein[a]: inheritance and relationship of their molecular weights to plasma levels of lipoprotein[a]. J Lipid Res.

[39] Erqou S, Thompson A, Di Angelantonio E, Saleheen D, Kaptoge S, Marcovina S, Danesh J (2010). Apolipoprotein(a) isoforms and the risk of vascular disease: systematic review of 40 studies involving 58,000 participants. J Am Coll Cardiol.

[40] Levey AS, Stevens LA, Schmid CH, Zhang Y Lucy, Castro AF, Feldman HI, Kusek JW, Eggers P, Van Lente F, Greene T, Coresh J (2009). A new equation to estimate glomerular filtration rate. Ann Intern Med.

[41] Alkarkhi AFM, Alqaraghuli WAA, Alkarkhi AFM, Alqaraghuli WAA (2019). Chapter 8 - Principal Components Analysis.

[42] Viechtbauer W (2010). Conducting meta-analyses in R with the metafor package. J Stat Software.

[43] Ebbert MTW, Jensen TD, Jansen-West K, Sens JP, Reddy JS, Ridge PG, Kauwe JSK, Belzil V, Pregent L, Carrasquillo MM, Keene D (2019). Systematic analysis of dark and camouflaged genes reveals disease-relevant genes hiding in plain sight. Genome Biol.

[44] van der Harst P, Verweij N (2018). Identification of 64 novel genetic loci provides an expanded view on the genetic architecture of coronary artery disease. circ, Res.

[45] The UniProt Consortium (2021). UniProt: the universal protein knowledgebase in 2021. Nucleic Acids Res.

[46] Sandholzer C, Saha N, Kark JD, Rees A, Jaross W, Dieplinger H, Hoppichler F, Boerwinkle E, Utermann G (1992). Apo(a) isoforms predict risk for coronary heart disease. A study in six populations. Arterioscler Thromb A J Vasc Biol.

[47] Trinder M, Uddin MM, Finneran P, Aragam KG, Natarajan P (2021). Clinical utility of lipoprotein(a) and LPA genetic risk score in risk prediction of incident atherosclerotic cardiovascular disease. JAMA Cardiol.

[48] Puckey LH, Lawn RM, Knight BL (1997). Polymorphisms in the apolipoprotein(a) gene and their relationship to allele size and plasma lipoprotein(a) concentration. Hum Mol Genet.

[49] Lanktree MB, Rajakumar C, Brunt JH, Koschinsky ML, Connelly PW, Hegele RA (2009). Determination of lipoprotein(a) kringle repeat number from genomic DNA: copy number variation genotyping using qPCR. J Lipid Res.

[50] Lanktree MB, Anand SS, Yusuf S, Hegele RA (2010). Comprehensive analysis of genomic variation in the LPA locus and its relationship to plasma lipoprotein(a) in South Asians, Chinese, and European Caucasians. Circ Cardiovasc Genet.

[51] Burgess S, Ference BA, Staley JR, Freitag DF, Mason AM, Nielsen SF, Willeit P, Young R, Surendran P, Karthikeyan S, Bolton TR (2018). Association of LPA variants with risk of coronary disease and the implications for lipoprotein(a)-lowering therapies: a mendelian randomization analysis. JAMA Cardiol.

[52] Khera AV, Chaffin M, Aragam KG, Haas ME, Roselli C, Choi SH, Natarajan P, Lander ES, Lubitz SA, Ellinor PT, Kathiresan S (2018). Genome-wide polygenic scores for common diseases identify individuals with risk equivalent to monogenic mutations. Nat Genet.

[53] Dron JS, Wang M, Patel AP, Kartoun U, Ng K, Hegele RA, Khera AV (2021). Genetic predictor to identify individuals with high lipoprotein(a) concentrations. Circ Genomic Precis Med.

[54] Wu H, Luan J, Forgetta V, Engert JC, Thanassoulis G, Mooser V, Wareham NJ, Langenberg C, Richards JB (2021). Utility of genetically predicted lp(a) (lipoprotein [a]) and ApoB levels for cardiovascular risk assessment. circ, Genomic Precis Med.

