# Supplementary material for: The effect of LPA Thr3888Pro on lipoprotein(a) and coronary artery disease is modified by the LPA KIV-2 variant 4925G>A
Source: Atherosclerosis. Author manuscript; Available in PMC 2022 Sep 15. (PMC7613586; doi:10.1016/j.atherosclerosis.2022.04.023)
Supplement: Supplementary File [file EMS152739-supplement-Supplementary_File.pdf]

Supplemental materials to

The effect of *LPA* Thr3888Pro on lipoprotein(a) and coronary artery disease is modified by the *LPA* KIV-2 variant 4925G>A

Rebecca Grüneis, MSc[1], Claudia Lamina, PhD[1], Silvia Di Maio, MSc[1], Sebastian Schönherr, PhD[1], Peter Zoescher, MSc[1], Lukas Forer, PhD[1], Gertraud Streiter, BSc[1], Annette Peters, PhD[2,3], Christian Gieger, PhD[2,3,4], Anna Köttgen, MD, MPH[5], Florian Kronenberg, MD[1], Stefan Coassin, PhD[1]

[1] Institute of Genetic Epidemiology, Department of Genetics and Pharmacology, Medical University of Innsbruck

[2] German Center for Diabetes Research (DZD), München-Neuherberg, Germany

[3] Institute of Epidemiology, Helmholtz Zentrum München - German Research Center for Environmental Health, Neuherberg, Germany

[4] Research Unit of Molecular Epidemiology, Helmholtz Zentrum München, German Research Center for Environmental Health, Neuherberg, Germany

[5] Institute of Genetic Epidemiology, Faculty of Medicine and Medical Center, University of Freiburg, Freiburg, Germany and German Chronic Kidney Disease study, Germany

Supplementary Methods

Description of the GCKD study

The GCKD [1,2] study includes German participants from nine recruiting centers suffering of moderate chronic kidney disease (CKD). Moderate CKD is classified as CKD Stage 3 with an estimated glomerular filtration rate (eGFR) according to the CKD_EPI equation [3] of 30-60 mL/min per 1.73 m^2 or overt proteinuria and eGFR >60 mL/min per 1.73 m^2. Overt proteinuria shows an albumin to creatinine ration of >300 mg/g or a protein to creatinine ratio in 24h urine of >500 mg/g. Individuals with active malignancy, NYHA IV heart failure, renal or any other transplantation, non-Caucasian origin and legal attendance were excluded. Study approval was done by the review boards of the participating institutions and informed consent was obtained from all participants.

Description of the KORA F3 and F4 studies

The KORA [4] (Cooperative Health Research in the Augsburg region, Kooperative Gesundheitsforschung in der Region Augsburg) F3 and F4 cohorts are follow-up studies of the previous studies KORA S3 and S4 and represent the general population in Augsburg and surrounding counties (Southern Germany). KORA F3 and F4 do not overlap. Inclusion criteria are German nationality and aged 25 to 74. Recruiting time for KORA F3 was 2004 and 2005 (n=3,184), for KORA F4 2006 and 2008 (n=3,080). Lp(a) concentrations and apo(a) isoforms were assessed for 3,516 participants in the KORA F3 and for 3,161 participants in the KORA F4 cohort. Available DNA samples at our institute were 3,161 from KORA F3 and 3,063 from KORA F4.

Lp(a) phenotyping

Lp(a) concentration was determined in mg/dL by ELISA [5,6]. A polyclonal affinity-purified rabbit anti-human apo(a) antibody was used for coating and a horseradish peroxidase-conjugated monoclonal anti-apo(a) antibody 1A2 for detection [7]. OD was quantified on two dilutions per sample (1:150 and 1:1500) and measurements within the linear range of the 7-point standard curve were accepted.

Apo(a) isoforms were assessed by Western blotting [6,8]. 150 ng Lp(a) were loaded and separated on a 1.46% agarose gel with 0.08% SDS for 18 h at 0.04 A constant current. A size standard containing apo(a) isoform 13, 19, 23, 27 and 35 KIV repeats (validated by fiber-FISH [9]) was applied in every seventh well of the gel. The gel was semi-dry electro-blotted to a PVDF membrane. The membrane was blocked with 1% BSA, 85 mM NaCl, 10 mM TRIS, 0.2% Triton X-100 for 30 min at 37°C and

incubated with horseradish-peroxidase-conjugated 1A2 antibody. Signals were detected with ECL substrate (WesternBright Chemilumineszenz Spray, Biozym, Vienna, AT) and recorded on autoradiography films (Amersham Hyperfilm™ ECL™, GE Healthcare, Chicago, IL, USA). A detailed protocol for ELISA and Western blotting has been published in [8].

Analysis of UK Biobank data

Genotypes of rs41272110 were retrieved from microarray genotyping data and 4925G>A carrier status was retrieved from whole exome sequencing data (n=199,126) using sequencing data reanalysis strategies detailed before [10]. Sequencing data were downloaded as CRAM data (UKB Data Field 23153) and all reads from the *LPA* KIV-2 region were extracted as defined in the bed files of Ebbert et al [10] (https://github.com/mebbert/Dark_and_Camouflaged_genes). The extracted sequencing reads were realigned to a single KIV-2 copy number as reference [10–12] and 4925G>A was called using a standard second-generation variant caller (https://github.com/seppinho/mutserve) capable to detect variants down to 1% mutation level [12].

Data were restricted to Caucasians (British, Irish or any other white ethnic background) with available exome data for 4925G>A and genotype data for rs41272110 (n=186,088). The impact of 4925G>A and rs41272110 on Lp(a) concentrations was investigated by quantile regression in 173,878 participants. The first reported diagnosis (International Classification of Disease version 10 ICD-10 I21-25) [13,14] of coronary artery disease (CAD) was used for survival analysis. CAD included acute myocardial infarction, subsequent myocardial infarction, complications following acute myocardial infarction (UKB macrogroup “certain current complications following acute myocardial infarction”, I23.0, I23.1, I23.2, I23.3, I23.5, I23.6, I23.8), other acute ischaemic heart diseases and chronic ischaemic heart diseases. The first occurrence was provided by UKB by mapping self-report at any assessment centre, inpatient hospital data, primary care or death record data. Hazard ratio for CAD was estimated as a function of the carrier status of the two SNPs independently from each other, as well as a joint model (one variant adjusted for the other) and additionally adjusted for sex (n=186,088). Age was used as timescale, meaning that the observation period starts from the year of birth and censored the data as of 1st of January 2020, including 13,335 incident CAD events, independently of their SNPs carrier status. One individual was excluded due to an implausible date of CAD event. UKB analyses were performed in R version 3.6.3 and the R package *survival* was used for survival analysis.

Supplementary Figures

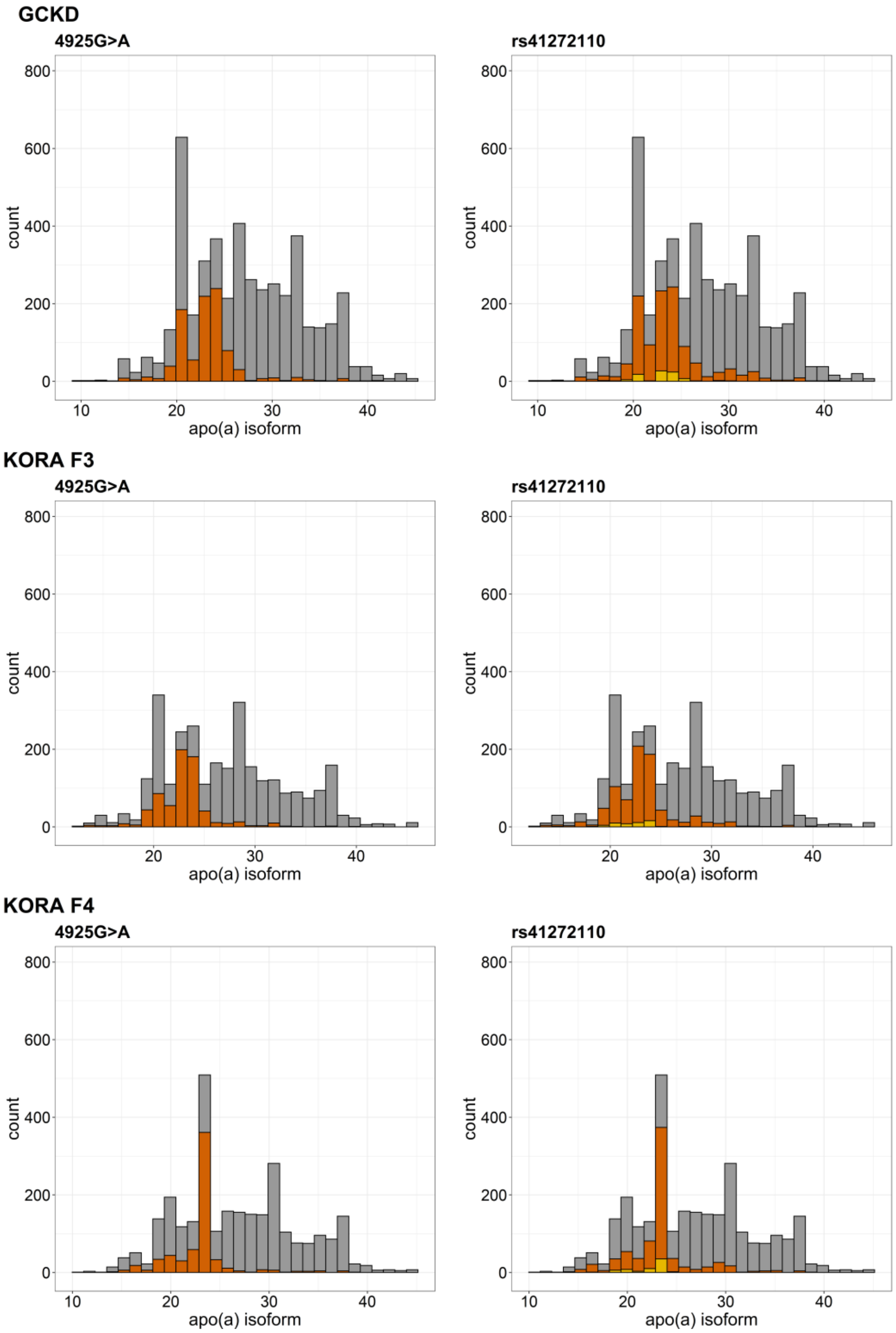

Supplementary Figure I. Isoform distribution of the variant carriers in the different studies.
Both variants are predominantly observed in isoforms with 20-25 KIV repeats in all three populations.

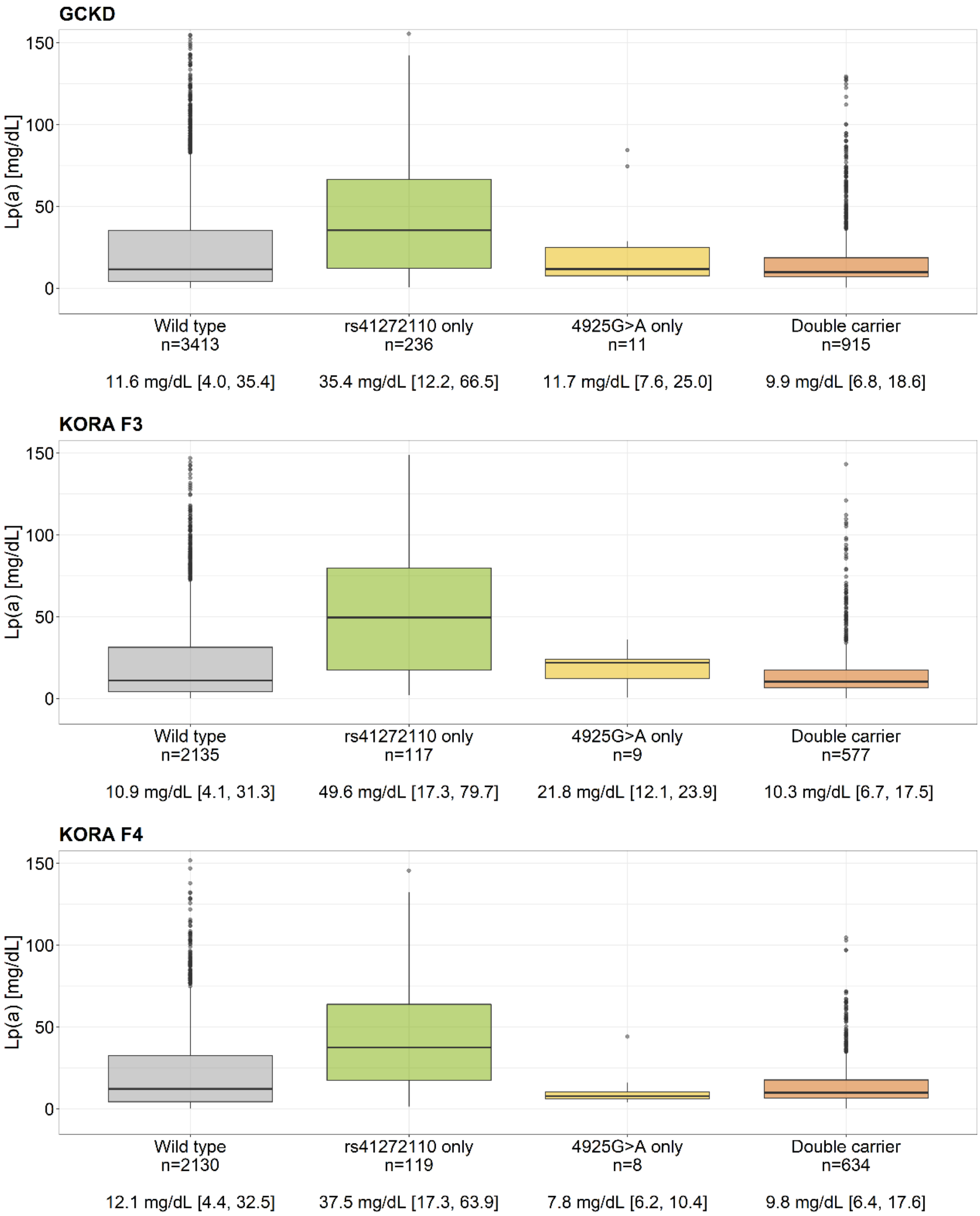

Supplementary Figure II. Lp(a) distribution in the four genotype combinations.

Lp(a) concentration of the different genotype combinations (wild type, rs41272110 only, 4925G>A only, double carriers) in GCKD, KORA F3 and KORA F4. Compared to wild type rs41272110 is significantly associated with increased Lp(a) concentrations, whereas no difference was observed between 4925G>A and double carriers. Since 4925G>A is associated with a defined isoform range [11], its effect becomes appreciable only after isoform stratification (Main Figure 1, Supplementary Figures III-IV). Median Lp(a) concentration and IQR for every genotype combination are given under the x-axis of the figure as well as in Supplementary Table IV. The box represents the IQR and the central line the median. Whiskers depict 1.5*IQR from the hinge.

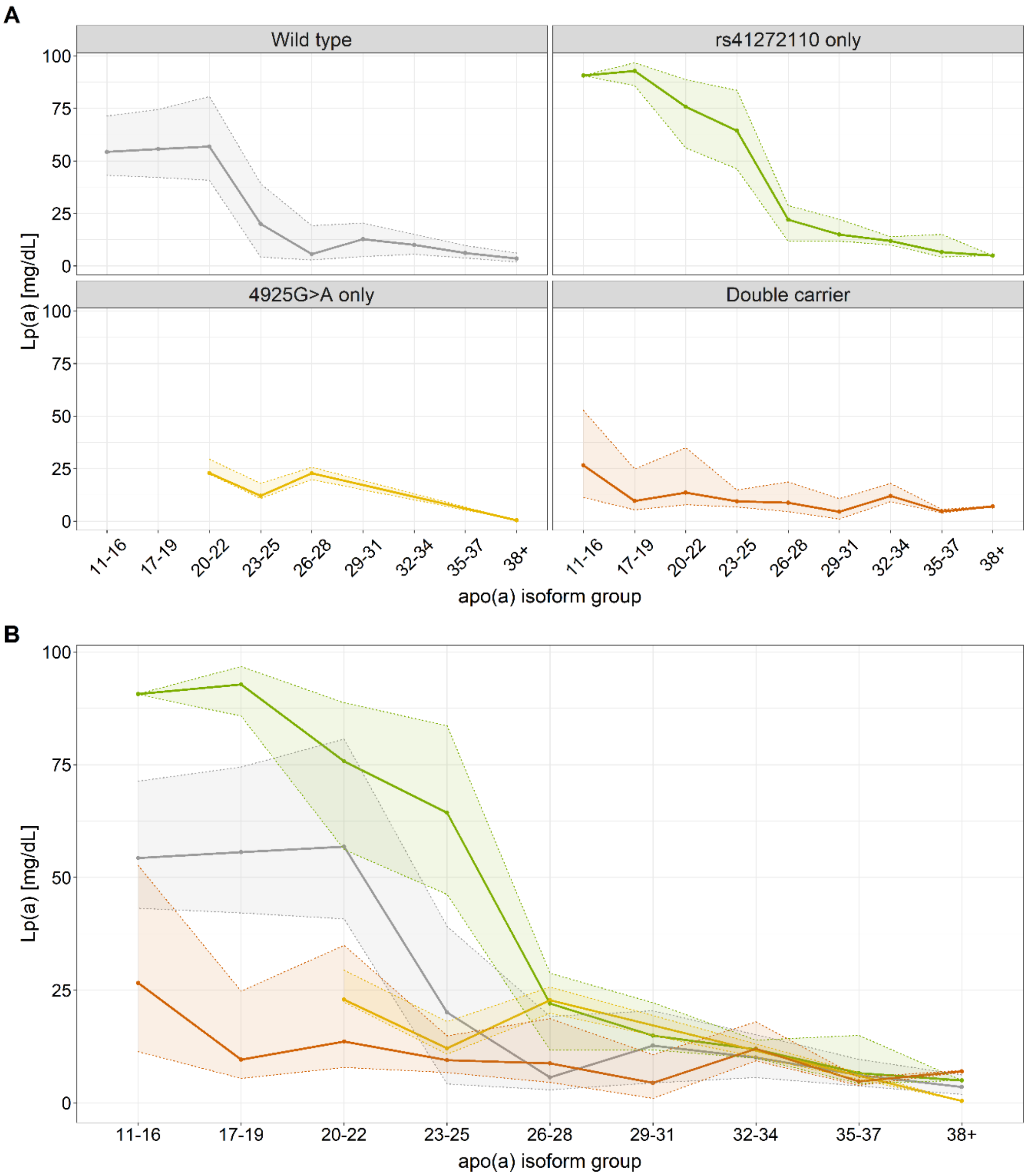

Supplementary Figure III. Median and interquartile Lp(a) concentration of the four genotype combinations over the whole isoform range in KORA F3.

(A) Single distributions of all genotype combinations in the KORA F3 study; (B) Superimposed Lp(a) distributions of all genotype combinations in the KORA F3 study; compared to wild type Lp(a) concentrations are reduced in double carriers but increased in individuals carrying rs41272110 only. Solid line represents the median Lp(a) concentration. Shaded area represents the interquartile range.

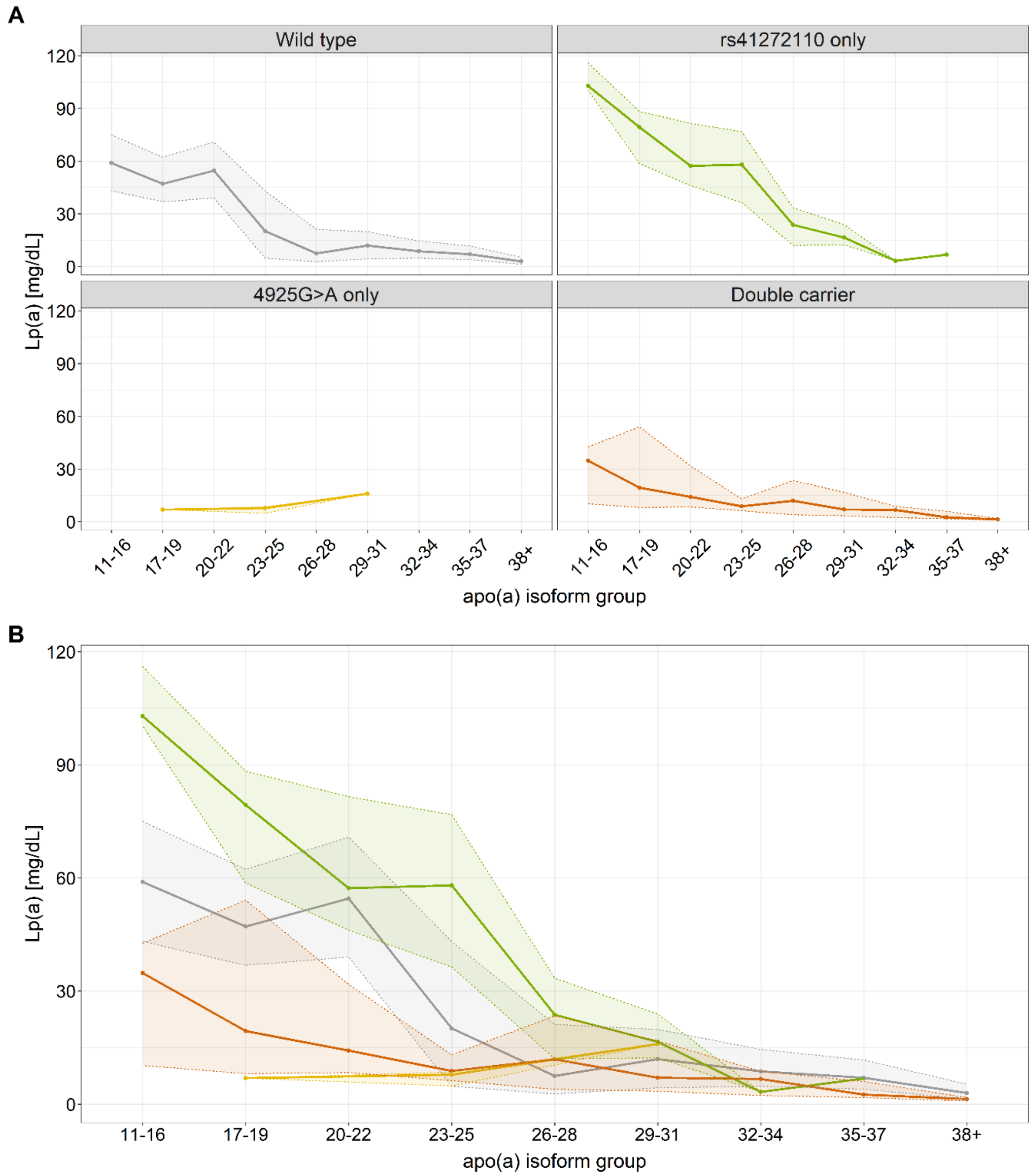

Supplementary Figure IV. Median and interquartile Lp(a) concentration of the four genotype combinations over the whole isoform range in KORA F4.

(A) Single distributions of all genotype combinations in the KORA F4 study; (B) Superimposed Lp(a) distributions of all genotype combinations in the KORA F4 study; compared to wild type Lp(a) concentrations are reduced in double carriers, but increased in individuals carrying rs41272110 only. Solid line represents the median Lp(a) concentration. Shaded area represents the interquartile range.

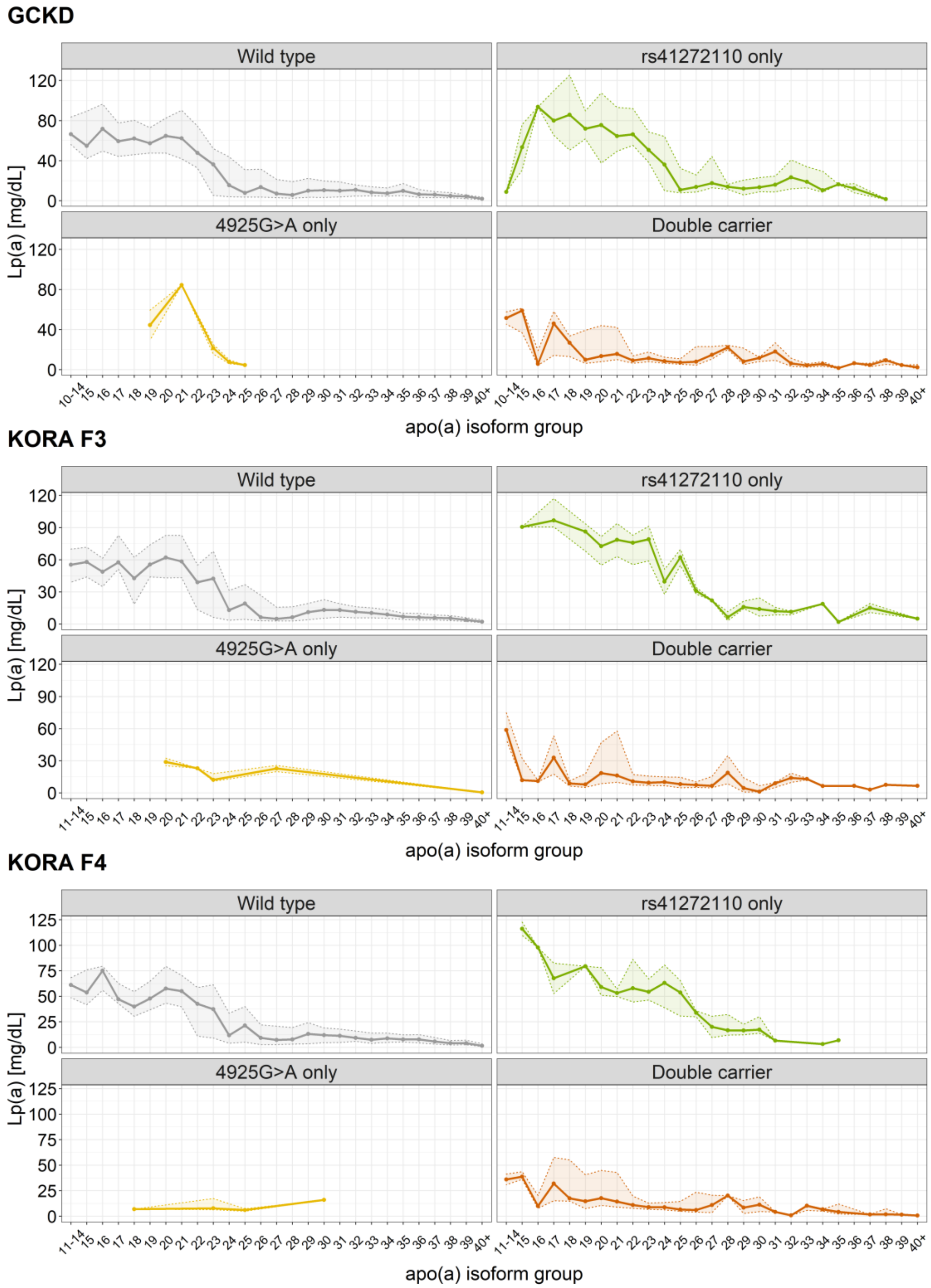

Supplementary Figure V. Median and interquartile Lp(a) concentration of the four genotype combinations for the single isoforms in all study populations.

Compared to wild type individuals, Lp(a) concentrations are reduced in double carriers, but increased in individuals carrying rs41272110 only. The solid line represents the median Lp(a) concentration. The shaded area represents the interquartile range.

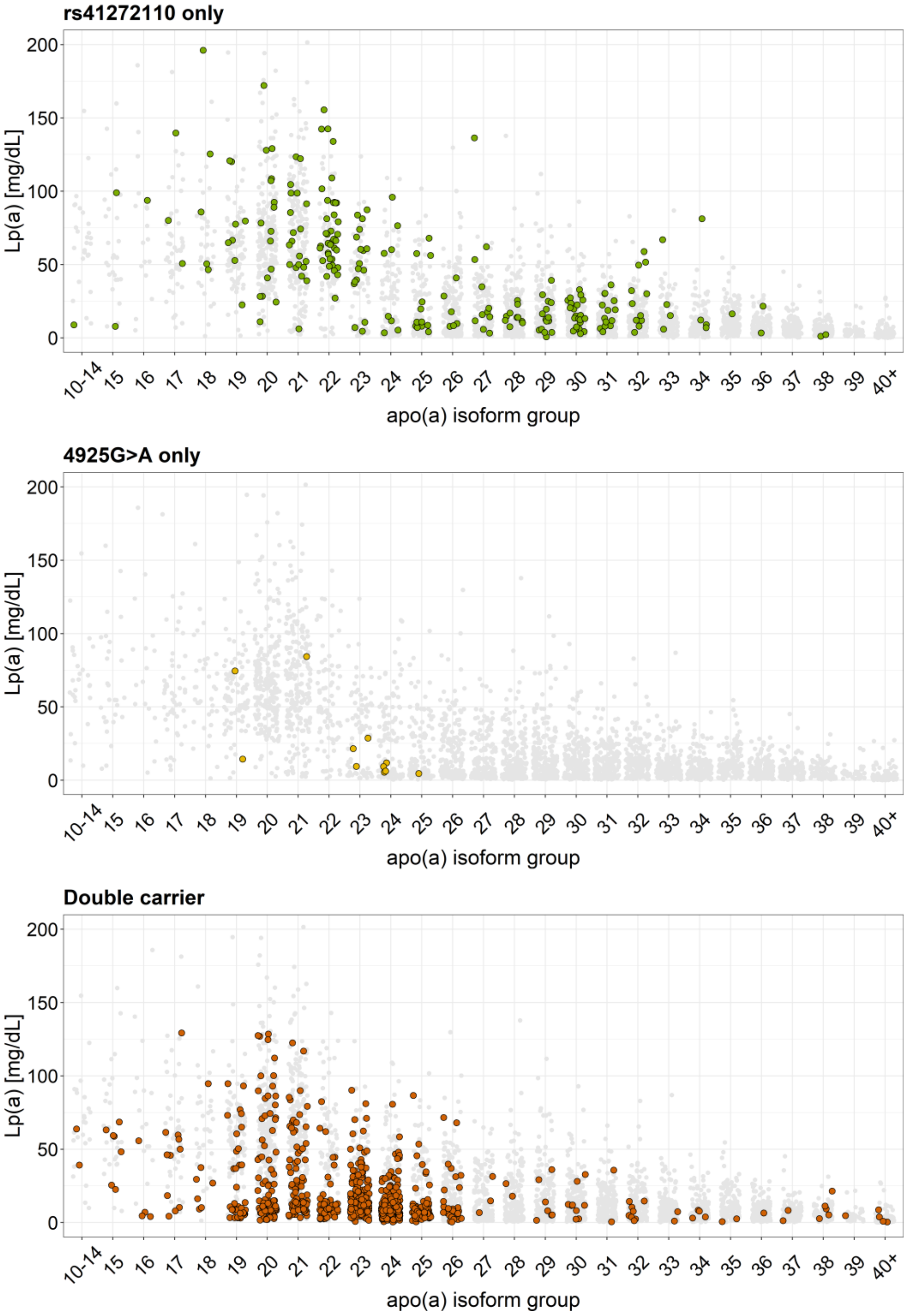

Supplementary Figure VI. Lp(a) concentration of the genotype combinations distributed over the different isoform groups in GCKD.

Carriers of rs41272110 only (green) show mainly high Lp(a) concentrations, whereas the few individuals carrying 4925G>A only (yellow) and double carriers (orange) show mainly low Lp(a) concentrations. Grey shaded individuals represent wild type individuals; y-scale is restricted to 200 mg/dL, 6 individuals negative for both variants are omitted.

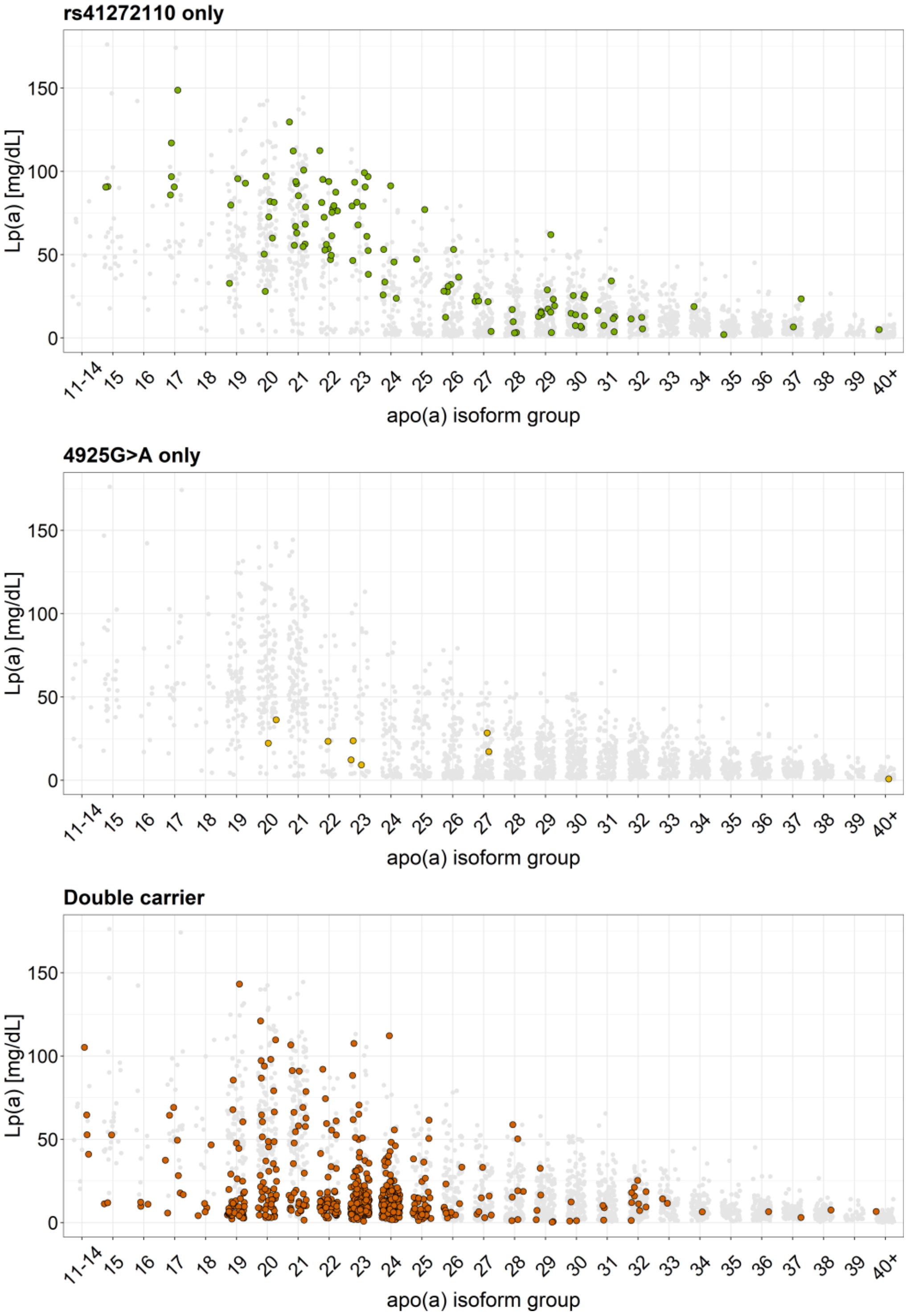

Supplementary Figure VII. Lp(a) concentration of the genotype combinations distributed over the different isoform groups in KORA F3.

Carriers of rs41272110 only (green) show mainly high Lp(a) concentrations, whereas the few individuals carrying 4925G>A only (yellow) and double carriers (orange) show mainly low Lp(a) concentrations. Grey shaded individuals represent wild type individuals.

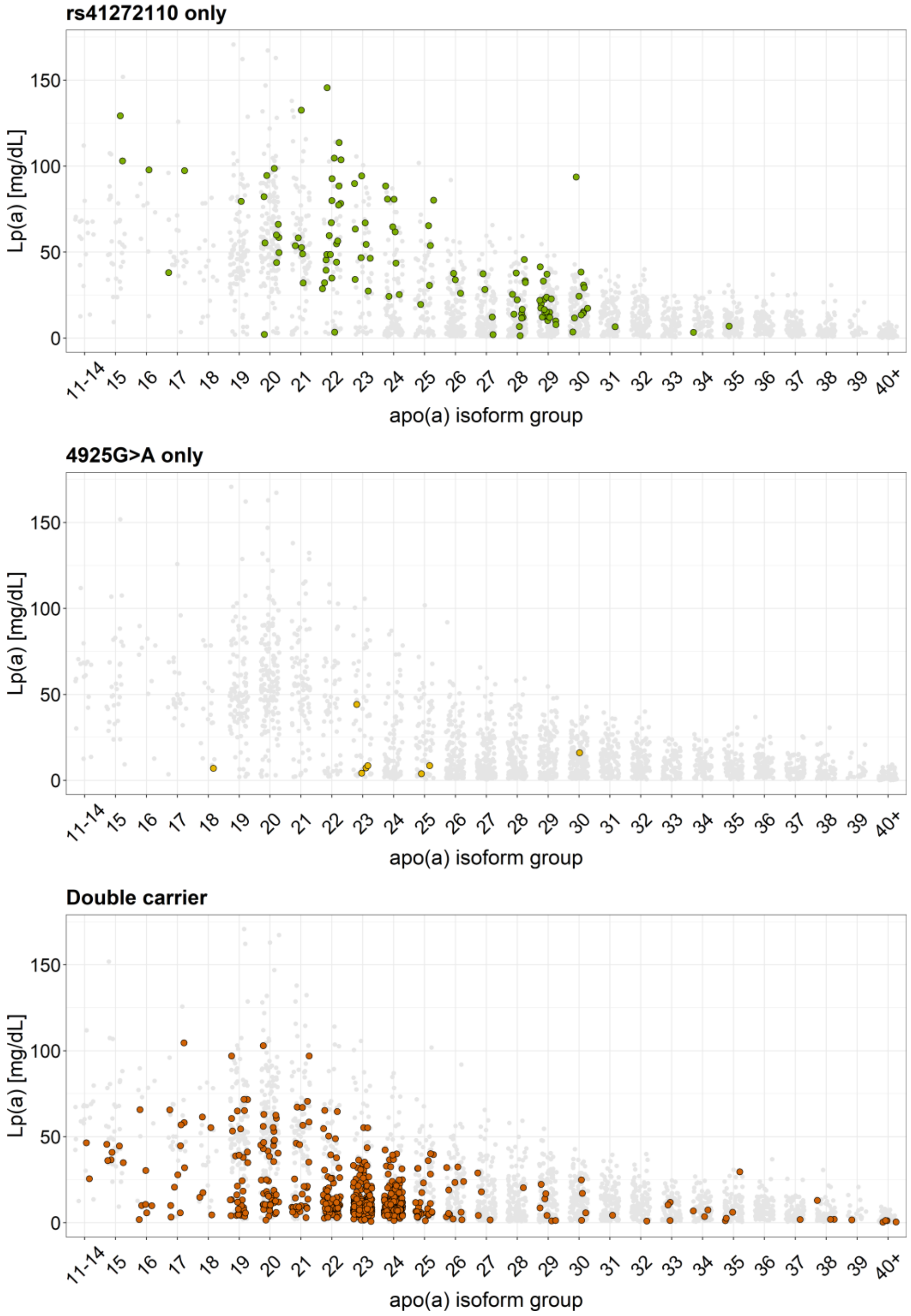

Supplementary Figure VIII. Lp(a) concentration of the genotype combinations distributed over the different isoform groups in KORA F4.

Carriers of rs41272110 only (green) show mainly high Lp(a) concentrations, whereas the few individuals carrying 4925G>A only (yellow) and double carriers (orange) show mainly low Lp(a) concentrations. Grey shaded individuals represent wild type individuals.

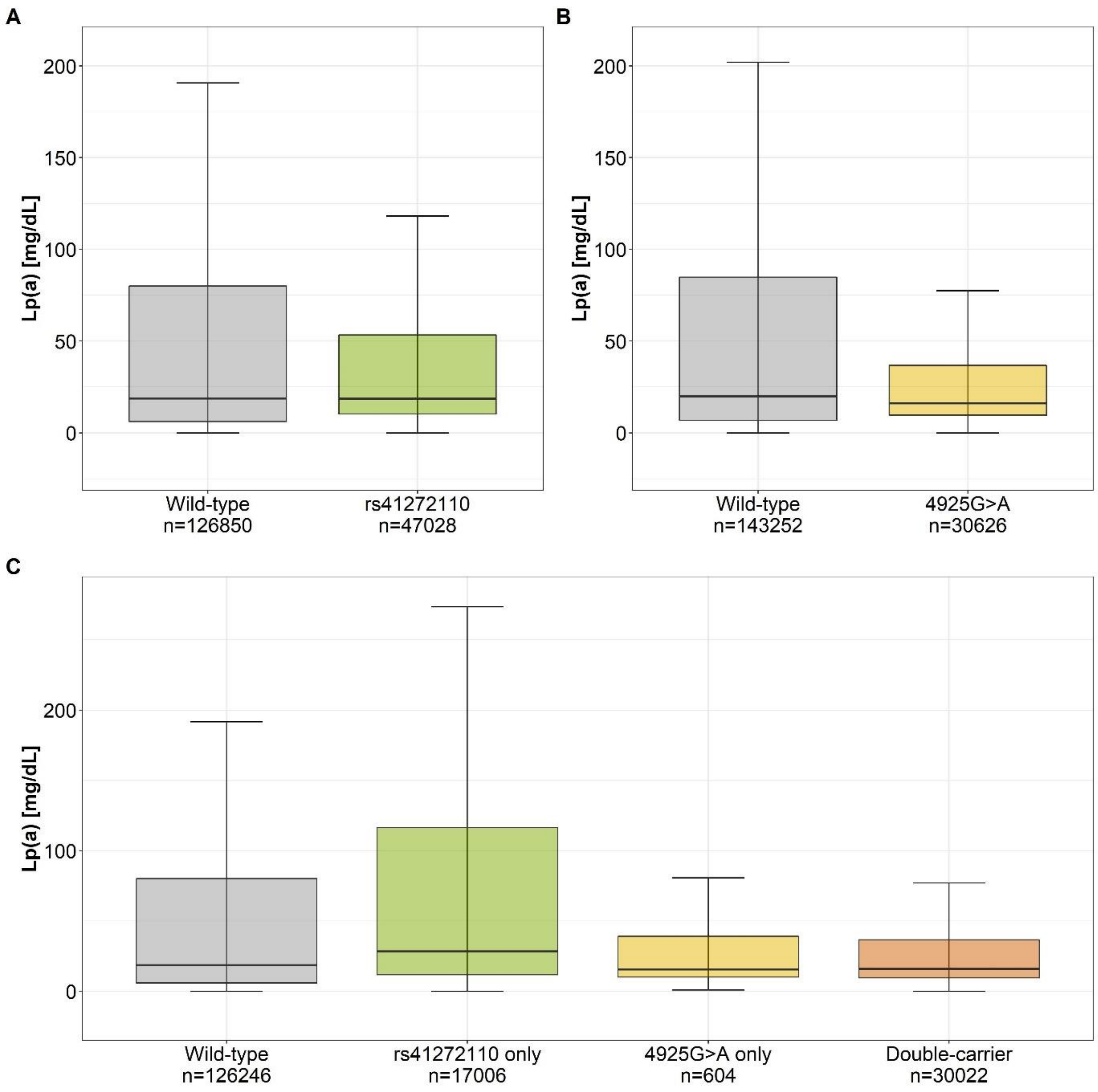

Supplementary Figure IX. Lp(a) distribution in the four genotype combinations in UK Biobank participants.

(A) rs41272110 carrier, (B) 4925G>A carrier, (C) different genotype combinations (wild type, rs41272110 only, 4925G>A only, double carrier). Median Lp(a) concentration and IQR for every genotype combination is shown in Supplementary Table IX. The box represents the IQR and the central line the median. Whiskers depict 1.5*IQR from the hinge.

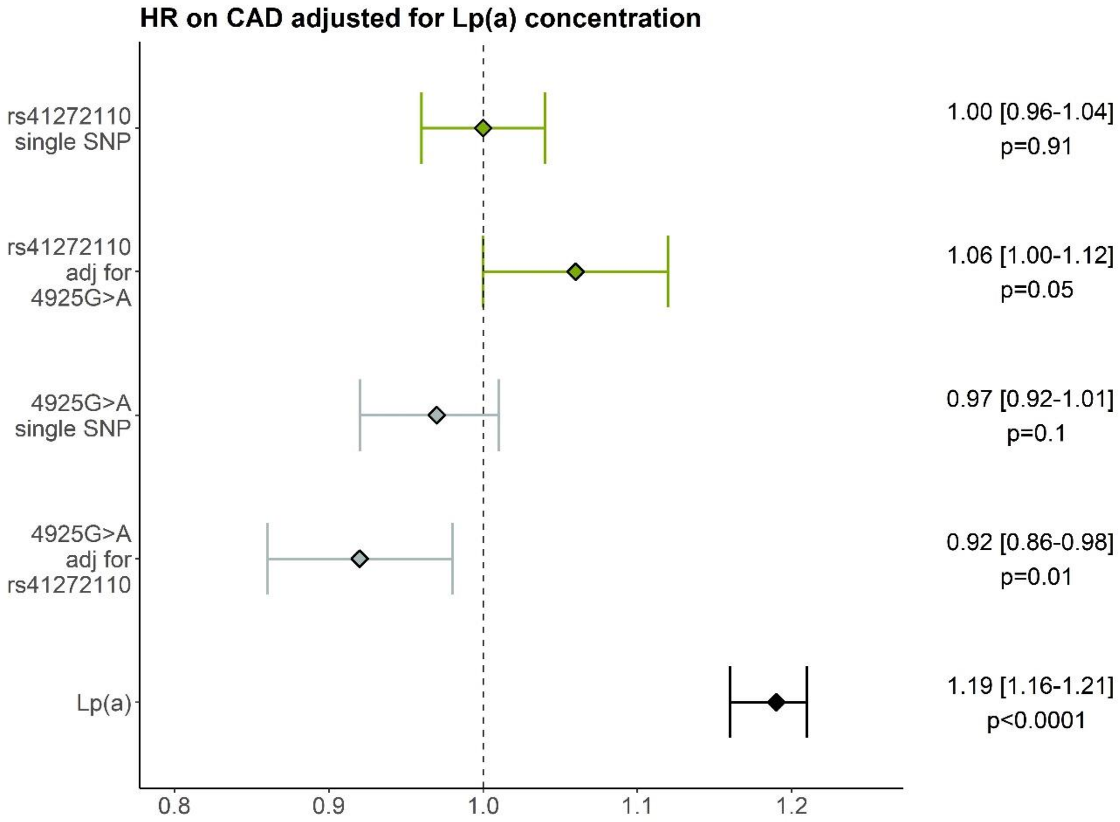

Supplementary Figure X. Impact of both variants on the CAD risk adjusted for Lipoprotein(a) concentrations.

The model is adjusted for sex and inverse-normal transformed Lp(a) concentration and restricted to individuals with Lp(a) measurements and data for both SNPs available (n=173,878). Age is taken as time scale and hazard ratio (HR) for Lp(a) is given for a 1-unit increase of the inverse-normal transformed Lp(a) concentration. In the joint model, the HR for CAD adjusted for Lp(a) was strongly alleviated and partially abolished. The HR of Lp(a) is highly significant and confirms the imperative role of the Lp(a) concentrations. adj.: adjusted

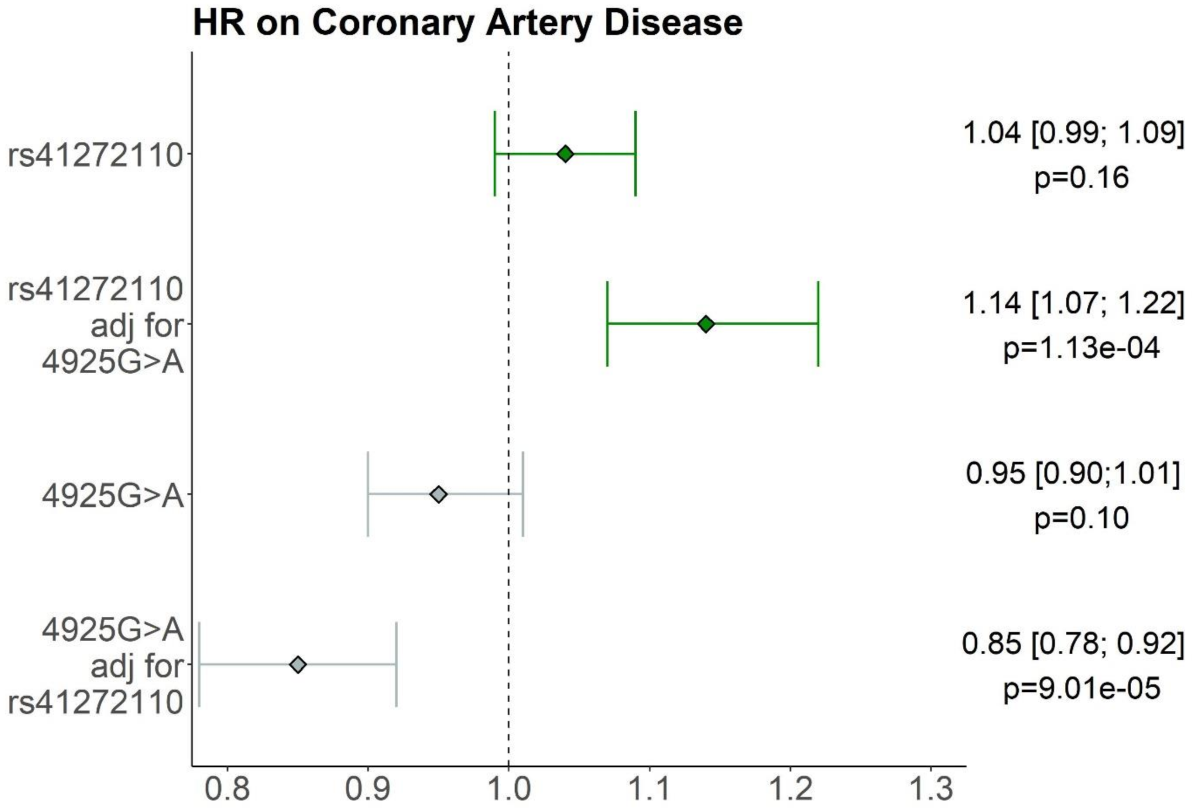

Supplementary Figure XI. Sensitivity analysis in UK Biobank: Hazard ratios (HR) of rs41272110 and 4925G>A in UK Biobank without genetic kinship to other participants (n=128,672). Rs41272110 (n=34,878) has no impact on CAD risk (total CAD events=8,922; 2,452 CAD events in rs41272110 carriers). 4925G>A carriers (n=22,373) show significantly reduced CAD risk (total CAD events=8,922; 1,480 CAD events in 4925G>A carriers). Conversely, in a joint model, hazard ratios show increased CAD risk in rs41272110 carriers (n=34,878) and reduced CAD risk in 4925G>A carriers (n=22,373). All Cox models for sensitivity analyses take participant age as timescale and are adjusted for sex and the first 30 principal components. adj.: adjusted

Supplementary Tables

Supplementary Table I. Study characteristics of previous investigations on rs41272110.

Study	Study population	n	Ancestry	Analysis stratified or adjusted for apo(a) isoforms	Effect on Lp(a)	Rs41272110 associated apo(a) isoform
**Prins et al. (1997)**[15]	153 subjects with symptomatic atherosclerosis, 153 controls	306	White European	no	decreasing	NR
**Prins et al. (1999)**[16]	healthy voluntary blood donors	201	White European	yes	increasing	23-29
**Ogorelkova et al. (2001)**[17]	non-related healthy subjects	130	White European (Tyrolean)	yes	decreasing	21-25
		104	White European (Finnish)	yes	increasing	21-25
**Chretien et al. (2006)**[18]	patients receiving incident dialysis (CHOICE cohort)	534	European Americans	yes	decreasing	17-21
		249	African Americans	yes	decreasing	NR
**Deo et al. (2011)**[19]	community-based observational study of cardiovascular disease (Jackson Heart Study)	4464	African Americans	no	no beta reported	NR
**Lee et al. (2016)**[20]	multiethnic, probability-based sample of the Dallas County population	1792	Black participants	yes	decreasing	NR
		1030	White participants	yes	increasing	NR
		597	Hispanic participants	yes	increasing	NR
**Yahya (2019)**[21]	dyslipidemia patients on statin treatment, mostly familial hypercholesterolemia	81	Primarily white European	no	no significant effect on Lp(a), but ~5 mg/dL higher median Lp(a) in carriers	NR

Supplementary Table II. Quantile regression analysis of rs41272110 and 4925G>A on Lp(a) concentration in GCKD, KORA F3 and KORA F4. In GCKD both models were further adjusted for eGFR.

				**Model 1: adjusted for age and sex**			**Model 2: adjusted for age, sex and smaller apo(a) isoform**		
**study**	**group**	**variant**	**carrier [n]**	**β [mg/dL]**	**95% CI [mg/dL]**	***p*-value**	**β [mg/dL]**	**95% CI [mg/dL]**	***p*-value**
**GCKD**	All	rs41272110	1,151	-0.13	-0.87, 0.61	0.733	-12.79	-14.41, -11.16	2.13E-52
		4925G>A	926	-2.47	-3.36, -1.58	5.93E-08	-22.67	-24.25, -21.09	2.67E-160
	LMW	rs41272110	402	-26.75	-32.93, -20.58	6.01E-17	-26.47	-32.40, -20.54	7.46E-18
		4925G>A	310	-48.51	-51.95, -45.08	3.80E-129	-47.65	-51.23, -44.07	6.14E-118
	HMW	rs41272110	749	+1.55	1.06, 2.03	4.14E-10	-1.15	-1.76, -0.54	0.0002
		4925G>A	616	+0.76	0.15, 1.38	0.015	-3.30	-4.29, -2.31	6.52E-11
**KORA F3**	All	rs41272110	795	+0.68	-0.19, 1.54	0.124	-12.49	-13.75, -11.23	1.75E-79
		4925G>A	687	-1.27	-2.37, -0.17	0.0243	-19.10	-20.87, -17.32	2.18E-92
	LMW	rs41272110	252	-27.08	-34.10, -20.07	1.23E-13	-26.95	-33.87, -20.03	7.92E-14
		4925G>A	208	-44.83	-48.49, -41.17	1.09E-92	-44.95	-48.73, -41.17	1.17E-88
	HMW	rs41272110	542	+2.42	1.67, 3.17	3.44E-10	-0.50	-1.38, 0.38	0.263
		4925G>A	478	+1.49	0.64, 2.33	0.000574	-2.86	-4.00, -1.72	8.78E-07
**KORA F4**	All	rs41272110	753	-0.64	-1.39, 0.11	0.0967	-11.34	-13.28, -9.40	1.20E-29
		4925G>A	642	-3.04	-4.10, -1.98	2.07E-08	-19.75	-21.29, -18.22	4.95E-127
	LMW	rs41272110	242	-21.07	-25.21, -16.94	4.53E-22	-20.01	-24.09, -15.93	1.30E-20
		4925G>A	199	-38.34	-42.27, -34.41	5.61E-66	-35.67	-39.83, -31.51	1.47E-53
	HMW	rs41272110	511	+1.19	0.52, 1.86	0.0005	-1.50	-2.44, -0.56	0.00187
		4925G>A	443	-0.41	-1.29, 0.47	0.359	-4.70	-5.97, -3.43	5.89E-13

Supplementary Table III. Sensitivity analysis: Quantile regression of rs41272110 and 4925G>A on Lp(a) concentration in a restricted dataset without relatives and adjusted for the first 10 (GCKD) and 8 (KORA F3) principal components. In GCKD both models were further adjusted for eGFR.

				**Model 1: adjusted for age and sex**			**Model 2: adjusted for age, sex and smaller apo(a) isoform**		
**study**	**group**	**variant**	**carrier [n]**	**β [mg/dL]**	**95% CI [mg/dL]**	***p*-value**	**β [mg/dL]**	**95% CI [mg/dL]**	***p*-value**
**GCKD**	All	rs41272110	1151	-0.26	-0.30, -0.21	0.438	-12.83	-14.50, -11.16	2.78E-50
		4925G>A	926	-2.38	-3.25, -1.52	7.56E-08	-22.53	-13.63, -11.00	1.58E-168
	LMW	rs41272110	402	-26.98	-32.71, -21.25	1.28E-19	-25.86	-31.04, -20.68	9.50E-22
		4925G>A	310	-47.71	-51.09, -44.32	8.32E-129	-47.16	-50.48, -43.83	5.09E-130
	HMW	rs41272110	749	+1.29	0.83, 1.75	4.77E-08	-1.19	-1.70, -0.67	7.19E-06
		4925G>A	616	+0.72	0.02, 0.10	0.022	-3.19	-4.17, -2.20	2.72E-10
**KORA F3**	All	rs41272110	750	+0.72	-0.09, 1.54	0.083	-12.32	-13.63, -11.01	1.24E-71
		4925G>A	649	-1.44	-2.50, -0.37	0.008	-19.01	-20.83, -17.20	2.61E-87
	LMW	rs41272110	238	-26.32	-32.80, -19.84	7.83E-15	-25.82	-32.50, -19.15	1.17E-13
		4925G>A	197	-44.21	-47.86, -40.55	9.99E-90	-43.75	-47.52, -39.99	1.04E-84
	HMW	rs41272110	511	+2.39	1.82, 2.95	2.47E-16	-0.87	-1.53, -0.21	0.009
		4925G>A	451	+1.38	0.52, 2.23	0.00161	-3.03	-4.19, -1.88	2.93E-07

Supplementary Table IV. Quantile regression analysis of rs41272110 and 4925G>A on Lp(a) concentration by adjusting one variant for the other (joint model) in GCKD, KORA F3 and KORA F4. In GCKD both models were further adjusted for eGFR.

				**Model 1: adjusted for age and sex**			**Model 2: adjusted for age, sex and smaller apo(a) isoform**		
**study**	**group**	**variant**	**carrier [n]**	**β [mg/dL]**	**95% CI [mg/dL]**	***p*-value**	**β [mg/dL]**	**95% CI [mg/dL]**	***p*-value**
**GCKD**	All	rs41272110	1,151	+8.36	3.90, 12.82	2.44E-04	+4.28	2.78, 5.79	2.50E-08
		4925G>A	926	-10.91	-15.46, -6.37	2.56E-06	-26.94	-28.85, -25.02	2.47E-155
	LMW	rs41272110	402	+3.30	-2.99, 9.59	0.304	+4.71	-1.92, 11.35	0.164
		4925G>A	310	-51.29	-58.09, -44.48	2.77E-45	-51.96	-59.05, -44.88	3.92E-43
	HMW	rs41272110	749	+4.52	-10.59, 19.63	1.62E-14	+4.09	2.96, 5.21	1.20E-12
		4925G>A	616	-3.80	-5.12, -2.49	1.58E-08	-7.10	-8.64, -5.57	1.97E-19
**KORA F3**	All	rs41272110	795	+11.48	5.78, 17.17	7.95E-05	+5.48	2.15, 8.82	0.00126
		4925G>A	687	-12.99	-18.78, -7.20	1.15E-05	-24.61	-28.29, -20.92	4.56E-38
	LMW	rs41272110	252	+9.93	6.01, 13.85	8.69E-07	+10.91	6.02, 15.80	1.43E-05
		4925G>A	208	-54.56	-59.55, -49.58	2.58E-78	-55.29	-61.42, -49.17	6.34E-58
	HMW	rs41272110	542	+7.58	5.80, 9.35	1.14E-16	+6.02	3.86, 8.17	4.83E-08
		4925G>A	478	-6.44	-8.25, -4.64	3.65E-12	-8.88	-11.26, -6.49	3.78E-13
**KORA F4**	All	rs41272110	753	+9.03	3.63, 14.42	0.00105	+4.22	1.38, 7.07	3.64E-03
		4925G>A	642	-12.99	-18.5, -7.48	8.33E-06	-23.92	-27.12, -20.72	5.67E-47
	LMW	rs41272110	242	+1.75	-3.56, 7.06	0.519	+4.78	-1.35, 10.92	0.127
		4925G>A	199	-40.01	-46.98, -33.03	5.04E-27	-41.16	-48.82, -33.50	3.44E-24
	HMW	rs41272110	511	+7.51	3.98, 11.03	3.14E-05	+5.87	3.98, 7.76	1.35E-09
		4925G>A	443	-7.93	-11.58, -4.27	2.25E-05	-10.48	-12.62, -8.33	3.09E-21

Supplementary Table V. Median Lp(a) concentration and IQR of the different genotype combinations in GCKD, KORA F3 and KORA F4. Populations were stratified into four genotype combination categories: individuals carrying only rs41272110, carrying only 4925G>A, carrying both variants (double carriers) and carrying neither of both (wild type).

study	Genotype combiantion	n	n (%) with Lp(a) concentration >50 mg/dL	median Lp(a) [mg/dL]	IQR [mg/dL]
**GCKD**	Wild type	3,413	586 (17.2)	11.58	3.96, 35.44
	rs41272110 only	236	94 (39.8)	35.44	12.24, 66.52
	4925G>A only	11	2 (18.2)	11.66	7.64, 25.04
	Double carriers	915	75 (8.2)	9.87	6.82, 18.54
**KORA F3**	Wild type	2,135	310 (14.5)	10.92	4.09, 31.27
	rs41272110 only	117	58 (49.6)	49.55	17.31, 79.70
	4925G>A only	9	0 (0)	21.81	12.10, 23.92
	Double carriers	678	50 (7.4)	10.29	6.71, 17.48
**KORA F4**	Wild type	2,130	294 (13.8)	12.11	4.38, 32.54
	rs41272110 only	119	43 (36.1)	37.52	17.32, 63.93
	4925G>A only	8	0 (0)	7.82	6.21, 10.36
	Double carriers	634	34 (5.4)	9.80	6.34, 17.63

Supplementary Table VI. Quantile regression analysis of different genotype combinations on Lp(a) concentration in GCKD, KORA F3 and KORA F4. Populations were stratified into four genotype combination categories: individuals carrying only rs41272110, carrying only 4925G>A, carrying both variants (double carriers) and carrying neither of both (wild type). The wild type group is the reference group in the regression model. Effect given in mg/dL. Table continues on next page. In GCKD both models were further adjusted for eGFR.

				Model 1: adjusted for age and sex			Model 2: adjusted for age, sex and smaller apo(a) isoform		
**study**	**group**	**Genotype combination**	**carrier [n]**	**β [mg/dL]**	**95% CI [mg/dL]**	**p-value**	**β [mg/dL]**	**95% CI [mg/dL]**	**p-value**
**GCKD**	All	Wild type	3,413	reference					
		rs41272110 only	236	24.41	22.37, 26.45	5.53E-115	9.93	7.78, 12.07	1.63E-19
		4925G>A only	11	0.57	-8.56, 9.70	0.903	-20.12	-29.66, -10.58	3.61E-05
		Double carriers	915	-1.84	-2.96, -0.71	0.0014	-21.93	-23.19, -20.68	6.28E-230
	LMW	Wild type	726	reference					
		rs41272110 only	95	8.22	2.94, 13.50	0.0023	9.65	4.13, 15.15	0.0006
		4925G>A only	3	NA	NA	NA	NA	NA	NA
		Double carriers	307	-47.74	-51.04, -44.44	4.44E-134	-46.6	-50.03, -43.15	5.89E-121
	HMW	Wild type	2,687	reference					
		rs41272110 only	141	7.44	5.86, 9.02	4.84E-20	5.93	4.29, 7.55	1.40E-12
		4925G>A only	8	1.7	-4.75, 8.16	0.605	-1.52	-8.17, 5.12	0.654
		Double carriers	608	1.02	0.20, 1.84	0.0149	-2.71	-3.66, -1.75	2.80E-08
**KORA F3**	All	Wild type	2,135	reference					
		rs41272110 only	117	38.25	35.53, 40.98	3.19E-148	19.83	17.50, 22.17	1.24E-59
		4925G>A only	9	10.31	0.72, 19.91	0.035	-8.35	-16.49, -0.21	0.045
		Double carriers	678	-0.5	-1.77, 0.77	0.441	-18.48	-19.64, -17.32	2.25E-184
	LMW	Wild type	424	reference					
		rs41272110 only	47	20.08	13.31, 26.84	9.30E-09	22.09	14.94, 29.25	2.36E-09
		4925G>A only	3	NA	NA	NA	NA	NA	NA

		Double carriers	205	-42.74	-46.49, -38.99	1.98E-83	-42.04	-46.01, -38.08	1.35E-74
	HMW	Wild type	1,708	reference					
		rs41272110 only	70	14.69	8.87, 20.51	8.02E-07	11.58	9.38, 13.78	1.94E-24
		4925G>A only	6	5.15	1.69, 8.60	0.00351	1.03	-6.29, 8.36	0.782
		Double carriers	472	1.9	1.07, 2.73	8.22E-06	-2.46	-3.54, -1.38	8.10E-06
**KORA F4**	All	Wild type	2,130	reference					
		rs41272110 only	119	24.27	21.57, 26.97	4.30E-66	13.17	10.66, 15.68	1.99E-24
		4925G>A only	8	-5.56	-15.71, 4.59	0.283	-20.31	-29.68, -10.93	2.26E-05
		Double carriers	634	-2.27	-3.57, -0.97	0.0006	-18.99	-20.26, -17.72	6.39E-166
	LMW	Wild type	468	reference					
		rs41272110 only	44	7.29	-1.11, 15.69	0.0893	10.69	2.41, 18.97	0.012
		4925G>A only	1	NA	NA	NA	NA	NA	NA
		Double carriers	198	-37.41	-41.90, -32.91	5.55E-51	-34.66	-39.09, -30.23	5.01E-46
	HMW	Wild type	1,662	reference					
		rs41272110 only	75	16.59	14.27, 18.91	1.00E-42	13.44	11.21, 15.66	1.82E-31
		4925G>A only	7	-0.63	-8.08, 6.82	0.869	-5.15	-12.24, 1.94	0.154
		Double carriers	436	0.06	-1.00, 1.11	0.918	-3.84	-4.97, -2.71	3.31E-11

Supplementary Table VII. Quantile regression analysis of rs41272110 and 4925G>A on Lp(a) concentration in Caucasians from UK Biobank with available Lp(a) concentration, rs41272110 genotype carrier status and 4925G>A exome data (n=173,878). Model adjusted for age and sex.

**Variant**	**carrier [n]**	**β [nmol/L]**	**95% CI [nmol/L]**	***p*-value**
rs41272110	47,028	-0.03	-0.38, 0.32	0.860
4925G>A	30,693	-3.77	-4,08, -3.45	1.20e-119
**Joint model analysis (one variant adjusted for the other)**				
rs41272110	47,028	+7.95	7.25, 8.64	7.55e-112
4925G>A	30,693	-10.45	-11.13, -9.78	3.79e-202
**Different genotype combinations**				
rs41272110 only	17,006	+9.44	8.48, 10.40	1.63e-82
4925G>A only	604	-2.51	-3.23, -1.79	7.90e-12
Double carriers	30,022	-2.59	-2.91, -2.27	9.76e-56

Supplementary Table VIII. Sensitivity analysis in UK Biobank: Quantile regression analysis of rs41272110 and 4925G>A on Lp(a) concentration in Caucasians with available Lp(a) concentration, rs41272110 genotype carrier status, 4925G>A exome data and no genetic kinship to other participants found (n=120,228). Model adjusted for age, sex and the first 30 principal components.

Variant	carrier [n]	β [nmol/L]	95% CI [nmol/L]	*p*-value
rs41272110	32,637	-0.17	-0.57, 0.22	0.406
4925G>A	20,998	-3.88	-4.25, -3.52	1.47e-97
**Joint model analysis (one variant adjusted for the other)**				
rs41272110	32,637	+7.80	6.96, 8.64	7.19e-74
4925G>A	20,998	-10.50	-11.32, -9.68	3.90e-138
**Different genotype combinations**				
rs41272110 only	12,034	+9.10	8.03, 10.18	7.04e-62
4925G>A only	395	-3.10	-4.93, -1.27	8.87e-04
Double carriers	20,603	-2.74	-3.11, -2.37	3.50e-47

Supplementary Table IX. Median Lp(a) concentration and IQR of the different genotype combinations in UK Biobank. Populations were stratified into four genotype combination categories: individuals carrying only rs41272110, individuals carrying only 4925G>A, individuals carrying both variants (double carriers) and individuals carrying neither of both (wild type).

study	genotype combination	n	n (%) with Lp(a) concentration >120 nmol/L	median Lp(a) [nmol/L]	IQR [nmol/L]
**UK Biobank**	Wild type	126,246	22,588 (18.0)	18.70	6.10, 80.30
	rs41272110 only	17,006	4,134 (24.3)	28.40	11.90, 116.60
	4925G>A only	604	63 (10.4)	15.70	10.12, 39.12
	Double carriers	30,022	2814 (9.4)	16.08	9.60, 36.70

References

[1] K.-U. Eckardt, B. Bärthlein, S. Baid-Agrawal, A. Beck, M. Busch, F. Eitner, A.B. Ekici, J. Floege, O. Gefeller, H. Haller, R. Hilge, K.F. Hilgers, J.T. Kielstein, V. Krane, A. Köttgen, F. Kronenberg, P. Oefner, H.-U. Prokosch, A. Reis, M. Schmid, E. Schaeffner, U.T. Schultheiss, S.A. Seuchter, T. Sitter, C. Sommerer, G. Walz, C. Wanner, G. Wolf, M. Zeier, S. Titze, The German Chronic Kidney Disease (GCKD) study: design and methods, Nephrol. Dial. Transplant. 27 (2012) 1454–1460. https://doi.org/10.1093/ndt/gfr456.

[2] S. Titze, M. Schmid, A. Köttgen, M. Busch, J. Floege, C. Wanner, F. Kronenberg, K.-U. Eckardt, for the G. study investigators, K.-U. Eckardt, S. Titze, H.-U. Prokosch, B. Bärthlein, A. Beck, T. Ganslandt, O. Gefeller, M. Schmid, J. Köster, M. Malzer, G. Schlieper, F. Eitner, S. Meisen, K. Kehl, E. Arweiler, J. Floege, E. Schaeffner, S. Baid-Agrawal, R. Schindler, S. Titze, S. Hübner, T. Dienemann, K.F. Hilgers, K.-U. Eckardt, A. Köttgen, U. Schultheiß, G. Walz, J.T. Kielstein, J. Lorenzen, H. Haller, C. Sommerer, M. Zeier, M. Busch, K. Paul, G. Wolf, R. Hilge, T. Sitter, V. Krane, D. Schmiedeke, S. Toncar, C. Wanner, A.B. Ekici, A. Reis, L. Forer, S. Schönherr, H. Weissensteiner, B. Kollertits, J. Raschenberger, F. Kronenberg, W. Gronwald, H. Zacharias, P. Oefner, for the G. study investigators, Disease burden and risk profile in referred patients with moderate chronic kidney disease: composition of the German Chronic Kidney Disease (GCKD) cohort, Nephrol. Dial. Transplant. 30 (2015) 441–451. https://doi.org/10.1093/ndt/gfu294.

[3] A.S. Levey, L.A. Stevens, C.H. Schmid, Y. (Lucy) Zhang, A.F. Castro, H.I. Feldman, J.W. Kusek, P. Eggers, F. Van Lente, T. Greene, J. Coresh, A New Equation to Estimate Glomerular Filtration Rate, Ann. Intern. Med. 150 (2009) 604–612. https://doi.org/10.7326/0003-4819-150-9-200905050-00006.

[4] H.-E. Wichmann, C. Gieger, T. Illig, for the M.S. Group, KORA-gen - Resource for Population Genetics, Controls and a Broad Spectrum of Disease Phenotypes, Gesundheitswesen. 67 (2005) 26–30. https://doi.org/10.1055/s-2005-858226.

[5] G. Erhart, C. Lamina, T. Lehtimäki, P. Marques-Vidal, M. Kähönen, P. Vollenweider, O.T. Raitakari, G. Waeber, B. Thorand, K. Strauch, C. Gieger, T. Meitinger, A. Peters, F. Kronenberg, S. Coassin, Genetic Factors Explain a Major Fraction of the 50% Lower Lipoprotein(a) Concentrations in Finns., Arterioscler. Thromb. Vasc. Biol. 38 (2018) 1230–1241. https://doi.org/10.1161/ATVBAHA.118.310865.

[6] F. Kronenberg, E. Kuen, E. Ritz, R. Junker, P. Konig, G. Kraatz, K. Lhotta, J.F. Mann, G.A. Muller, U. Neyer, W. Riegel, P. Reigler, V. Schwenger, A. Von Eckardstein, P. König, G. Kraatz, K. Lhotta, J.F. Mann, G.A. Müller, U. Neyer, W. Riegel, P. Reigler, V. Schwenger, A. Von Eckardstein, P. Konig, G. Kraatz, K. Lhotta, J.F. Mann, G.A. Muller, U. Neyer, W. Riegel, P. Reigler, V. Schwenger, A. Von Eckardstein, P. König, G. Kraatz, K. Lhotta, J.F. Mann, G.A. Müller, U. Neyer, W. Riegel, P. Reigler, V. Schwenger, A. Von Eckardstein, P. Konig, G. Kraatz, K. Lhotta, J.F. Mann, G.A. Muller, U. Neyer, W. Riegel, P. Reigler, V. Schwenger, A. Von Eckardstein, Lipoprotein(a) serum concentrations and apolipoprotein(a) phenotypes in mild and moderate renal failure., J. Am. Soc. Nephrol. 11 (2000) 105–15. https://doi.org/10.1681/ASN.V111105.

[7] H. Dieplinger, G. Gruber, K. Krasznai, S. Reschauer, C. Seidel, G. Burns, H.J. Müller, A. Császár, W. Vogel, H. Robenek, Kringle 4 of human apolipoprotein[a] shares a linear antigenic site with human catalase., J. Lipid Res. 36 (1995) 813–822. https://doi.org/10.1016/S0022-2275(20)40065-3.

[8] G. Erhart, C. Lamina, T. Lehtimäki, P. Marques-Vidal, M. Kähönen, P. Vollenweider, O.T. Raitakari, G. Waeber, B. Thorand, K. Strauch, C. Gieger, T. Meitinger, A. Peters, F. Kronenberg, S. Coassin, Genetic Factors Explain a Major Fraction of the 50% Lower Lipoprotein(a) Concentrations in Finns., Arterioscler. Thromb. Vasc. Biol. 38 (2018) 1230–1241. https://doi.org/10.1161/ATVBAHA.118.310865.

[9] M. Erdel, M. Hubalek, A. Lingenhel, K. Kofler, H.C. Duba, G. Utermann, Counting the repetitive kringle-IV repeats in the gene encoding human apolipoprotein(a) by fibre-FISH, Nat Genet. 21 (1999) 357–358. https://doi.org/10.1038/7681.

[10] M.T.W. Ebbert, T.D. Jensen, K. Jansen-West, J.P. Sens, J.S. Reddy, P.G. Ridge, J.S.K. Kauwe, V. Belzil, L. Pregent, M.M. Carrasquillo, D. Keene, E. Larson, P. Crane, Y.W. Asmann, N. Ertekin-Taner, S.G. Younkin, O.A. Ross, R. Rademakers, L. Petrucelli, J.D. Fryer, Systematic analysis of dark and camouflaged genes reveals disease-relevant genes hiding in plain sight, Genome Biol. 20 (2019) 97. https://doi.org/10.1186/s13059-019-1707-2.

[11] S. Coassin, G. Erhart, H. Weissensteiner, M. Eca Guimarães de Araújo, C. Lamina, S. Schönherr, L. Forer, M. Haun, J.L. Losso, A. Köttgen, K. Schmidt, G. Utermann, A. Peters, C. Gieger, K. Strauch, A. Finkenstedt, R. Bale, H. Zoller, B. Paulweber, K.-U. Eckardt, A. Hüttenhofer, L.A. Huber, F. Kronenberg, A novel but frequent variant in LPA KIV-2 is associated with a pronounced Lp(a) and cardiovascular risk reduction, Eur. Heart J. 38 (2017) 1823–1831. https://doi.org/10.1093/eurheartj/ehx174.

[12] S. Coassin, S. Schönherr, H. Weissensteiner, G. Erhart, L. Forer, J.L. Losso, C. Lamina, M. Haun, G. Utermann, B. Paulweber, G. Specht, F. Kronenberg, A comprehensive map of single-base polymorphisms in the hypervariable LPA kringle IV type 2 copy number variation region, J. Lipid Res. 60 (2019) 186–199. https://doi.org/10.1194/jlr.M090381.

[13] P. van der Harst, N. Verweij, Identification of 64 Novel Genetic Loci Provides an Expanded View on the Genetic Architecture of Coronary Artery Disease, Circ. Res. 122 (2018) 433–443. https://doi.org/10.1161/CIRCRESAHA.117.312086.

[14] J.F. Schachtl-Riess, A. Kheirkhah, R. Grüneis, S. Di Maio, S. Schoenherr, G. Streiter, J.L. Losso, B. Paulweber, K.-U. Eckardt, A. Köttgen, C. Lamina, F. Kronenberg, S. Coassin, K.-U. Eckardt, H. Meiselbach, M.P. Schneider, M. Schiffer, H.-U. Prokosch, B. Bärthlein, A. Beck, A. Reis, A.B. Ekici, S. Becker, D. Becker-Grosspitsch, U. Alberth-Schmidt, B. Hausknecht, A. Weigel, G. Walz, A. Köttgen, U.T. Schultheiß, F. Kotsis, S. Meder, E. Mitsch, U. Reinhard, J. Floege, T. Saritas, E. Schaeffner, S. Baid-Agrawal, K. Theisen, H. Haller, J. Menne, M. Zeier, C. Sommerer, J. Theilinger, G. Wolf, M. Busch, R. Paul, T. Sitter, C. Wanner, V. Krane, A. Börner-Klein, B. Bauer, F. Kronenberg, J. Raschenberger, B. Kollerits, L. Forer, S. Schönherr, H. Weissensteiner, P. Oefner, W. Gronwald, M. Schmid, J. Nadal, Frequent LPA KIV-2 Variants Lower Lipoprotein(a) Concentrations and Protect Against Coronary Artery Disease, J. Am. Coll. Cardiol. 78 (2021) 437–449. https://doi.org/10.1016/j.jacc.2021.05.037.

[15] J. Prins, F.R. Leus, Y.Y. Van Der Hoek, J.J.P. Kastelein, B.N. Bouma, H.J.M. Van Rijn, The identification and significance of a Thr→Pro polymorphism in kringle IV type 8 of apolipoprotein(a), Thromb. Haemost. 77 (1997) 949–954. https://doi.org/10.1055/s-0038-1656083.

[16] J. Prins, F.R. Leus, B.N. Bouma, H.J. van Rijn, The identification of polymorphisms in the coding region of the apolipoprotein (a) gene--association with earlier identified polymorphic sites and influence on the lipoprotein (a) concentration., Thromb. Haemost. 82 (1999) 1709–17. https://doi.org/10.1055/s-0037-1614903.

[17] M. Ogorelkova, Single nucleotide polymorphisms in exons of the apo(a) kringles IV types 6 to 10 domain affect Lp(a) plasma concentrations and have different patterns in Africans and Caucasians, Hum. Mol. Genet. 10 (2001) 815–824. https://doi.org/10.1093/hmg/10.8.815.

[18] J.P. Chretien, J. Coresh, Y. Berthier-Schaad, W.H.L. Kao, N.E. Fink, M.J. Klag, S.M. Marcovina, F. Giaculli, M.W. Smith, Three single-nucleotide polymorphisms in LPA account for most of the increase in lipoprotein(a) level elevation in African Americans compared with European Americans, J. Med. Genet. 43 (2006) 917–923. https://doi.org/10.1136/jmg.2006.042119.

[19] R.C. Deo, J.G. Wilson, C. Xing, K. Lawson, W.H.L. Kao, D. Reich, A. Tandon, E. Akylbekova, N.

Patterson, T.H. Mosley Jr, E. Boerwinkle, H.A. Taylor Jr, Single-Nucleotide Polymorphisms in LPA Explain Most of the Ancestry-Specific Variation in Lp(a) Levels in African Americans, PLoS One. 6 (2011) e14581. https://doi.org/10.1371/journal.pone.0014581.

[20] S.-R. Lee, A. Prasad, Y.-S. Choi, C. Xing, P. Clopton, J.L. Witztum, S. Tsimikas, LPA Gene, Ethnicity, and Cardiovascular Events, Circulation. 135 (2017) 251–263. https://doi.org/10.1161/CIRCULATIONAHA.116.024611.

[21] R. Yahya, K. Berk, A. Verhoeven, S. Bos, L. van der Zee, J. Touw, G. Erhart, F. Kronenberg, R. Timman, E. Sijbrands, J. Roeters van Lennep, M. Mulder, Statin treatment increases lipoprotein(a) levels in subjects with low molecular weight apolipoprotein(a) phenotype, Atherosclerosis. 289 (2019) 201–205. https://doi.org/10.1016/j.atherosclerosis.2019.07.001.
